# HIV-1 gp120 Promotes Lysosomal Exocytosis in Human Schwann Cells

**DOI:** 10.3389/fncel.2019.00329

**Published:** 2019-07-17

**Authors:** Gaurav Datta, Nicole M. Miller, Zahra Afghah, Jonathan D. Geiger, Xuesong Chen

**Affiliations:** Department of Biomedical Sciences, University of North Dakota School of Medicine and Health Sciences, Grand Forks, ND, United States

**Keywords:** gp120, lysosome exocytosis, P2X4, ATP, Schwann cell, DRG neuron

## Abstract

Human immunodeficiency virus type 1 (HIV-1) associated neuropathy is the most common neurological complication of HIV-1, with debilitating pain affecting the quality of life. HIV-1 gp120 plays an important role in the pathogenesis of HIV neuropathy via direct neurotoxic effects or indirect pro-inflammatory responses. Studies have shown that gp120-induced release of mediators from Schwann cells induce CCR5-dependent DRG neurotoxicity, however, CCR5 antagonists failed to improve pain in HIV- infected individuals. Thus, there is an urgent need for a better understanding of neuropathic pain pathogenesis and developing effective therapeutic strategies. Because lysosomal exocytosis in Schwann cells is an indispensable process for regulating myelination and demyelination, we determined the extent to which gp120 affected lysosomal exocytosis in human Schwann cells. We demonstrated that gp120 promoted the movement of lysosomes toward plasma membranes, induced lysosomal exocytosis, and increased the release of ATP into the extracellular media. Mechanistically, we demonstrated lysosome de-acidification, and activation of P2X4 and VNUT to underlie gp120-induced lysosome exocytosis. Functionally, we demonstrated that gp120-induced lysosome exocytosis and release of ATP from Schwann cells leads to increases in intracellular calcium and generation of cytosolic reactive oxygen species in DRG neurons. Our results suggest that gp120-induced lysosome exocytosis and release of ATP from Schwann cells and DRG neurons contribute to the pathogenesis of HIV-1 associated neuropathy.

## Introduction

Schwann cells are the most abundant glial cells in the peripheral nervous system, ensheathing all axons of peripheral nerves as either myelinating or non-myelinating cells. In addition to its role as insulators of axons, Schwann cells are crucial for the proper function and maintenance of peripheral nerves by providing metabolic and/or trophic support (Beirowski et al., 2014; Feldman et al., 2017; Sasaki et al., 2018) and modulating responses to nerve injury (Jessen and Mirsky, 2016; Kim et al., 2018). Disrupting Schwann cell function can compromise glial–axon communication, nerve homeostasis, and ultimately lead to fiber loss, neurodegeneration, and pain. Although cellular and molecular mechanisms underlying communication between Schwann cells and dorsal root ganglia (DRG) neurons remain to be investigated, Schwann cell dysfunction could play a key role in the pathogenesis of peripheral neuropathy, a highly complex and prevalent disease affecting 2.4% of the general population. The prevalence of peripheral neuropathy increases with age, with 8% in individuals 55 years of age or older in Europe and 14.8% in individuals 40 years of age or older in United States (Martyn and Hughes, 1997; Gregg et al., 2004). The causes of peripheral neuropathy, which may be hereditary or iatrogenic from the toxicity of drugs given as part of antiretroviral or chemotherapy regiments, are often secondary to systemic illnesses including diabetes and infectious causes such as human immunodeficiency virus type 1 (HIV-1).

Human immunodeficiency virus type 1 infection affects 35 million people worldwide. Although combined anti-retroviral therapy (ART) successfully suppresses HIV-1 and dramatically increases the lifespan of HIV-infected individuals, neurological complication of HIV-1 infection persists. Between 30 and 60% of HIV-infected individuals develop HIV-associated distal symmetric polyneuropathy (DSP), a peripheral sensory neuropathy that is characterized clinically by numbness, tingling, pain, paresthesia, and allodynia that begin in distal lower extremities symmetrically and extend to more proximal areas and the upper extremities later in the disease (Centner et al., 2013). Pathologically, DSP is characterized by loss of intraepidermal nerve fibers, nerve fiber swelling, mononuclear inflammation, distal degeneration of long axons in a “dying back” pattern and concurrent damage to the DRG (Schütz and Robinson-Papp, 2013; Bilgrami and O’Keefe, 2014; Kaku and Simpson, 2014, Aziz-Donnelly and Harrison, 2017; Prior et al., 2018). The pathogenesis of DSP was originally attributed to neurotoxic effects of HIV-1 infection, but this is likely to be an indirect effect because evidence of viral infection of sensory neurons is lacking (Keswani et al., 2003; Melli et al., 2006). Since the introduction of ART, sensory neuropathy resulting from neurotoxic antiretrovirals, especially stavudine (d4T), didanosine (ddI), and zalcitabine (ddC), commonly referred to as d−drugs, has been recognized. However, in resource-rich countries where these neurotoxic drugs are no longer widely used, the rate of HIV-1 sensory neuropathies remains unsatisfactorily high (Ellis et al., 2008; Letendre et al., 2009). Furthermore, the prevalence of HIV-1 DSP was high before the introduction of highly active antiretroviral therapy (Hall et al., 1991; Fuller et al., 1993).

The pathogenesis of DSP remains elusive, but several underlying mechanisms have been proposed (Schütz and Robinson-Papp, 2013; Stavros and Simpson, 2014; Prior et al., 2018); peripheral nerve degeneration could result from HIV-induced dysfunction of macrophages that release inflammatory mediators, from age-related HIV-comorbidities such as diabetes, or from neurotoxicity of gp120, a glycoprotein on the HIV-1 envelope. Although gp120 is able to bind to DRG neurons and may have a direct neurotoxic effect (Apostolski et al., 1993; Melli et al., 2006; Wallace et al., 2007; Berth et al., 2016; Wenzel et al., 2017), Schwann cell-dependent DRG neurotoxicity of gp120 has also been implicated; gp120 can bind to chemokine receptors (CXCR4) of Schwann cells and the subsequent release of chemokines can induce apoptosis of DRG neurons via the activation of the C-C chemokine receptor type 5 (CCR5) (Keswani et al., 2003; Melli et al., 2006). However, vicriviroc, a CCR5 antagonist, did not improve pain compared to placebo in a trial of 118 patients with HIV DSP (Yeh et al., 2010). Thus, further mechanistic studies of HIV DSP are warranted.

Lysosomes are acidic organelles responsible for the degradation of macromolecules derived from endocytic and autophagic substrates. Besides their classical role in degradation, lysosomes have been implicated in intercellular communications via lysosome exocytosis; lysosomes can respond to extracellular stimuli by docking at the interior of the cell surface, fuse with the plasma membranes, and release their contents. In the nervous system, lysosomal exocytosis represents as a new pathway for gliotransmitters secreted from astrocytes (Zhang et al., 2007; Li et al., 2008; Liu et al., 2011). In Schwann cells, lysosome exocytosis has also been demonstrated (Chen et al., 2012; Shin et al., 2012; Jung et al., 2014; Su et al., 2019) and lysosome exocytosis can play an important role in regulating myelination and the release of ATP, an important mediator of peripheral pain via activation of purinergic receptors (Surprenant et al., 1996; Hattori and Gouaux, 2012; Yamashita et al., 2016; Jurga et al., 2017; Ying et al., 2017). Thus, disturbances in Schwann cell function could be an important underlying factor for axonal degeneration as occurs in HIV-1 neuropathy (Orita et al., 2013; Viader et al., 2013; Beirowski et al., 2014; Bacallao and Monje, 2015; Monk et al., 2015; Brosius Lutz et al., 2017).

Based on recent findings in non-neural cells that ATP is transported to lysosomes by the vesicular nucleotide transporter (VNUT), where it regulates lysosomal P2X4 function (Cao et al., 2014; Zhong et al., 2016; Moriyama and Nomura, 2017; Moriyama et al., 2017), we investigated the role of gp120 in regulating lysosome exocytosis in Schwann cells and subsequently affecting DRG neuron function. We demonstrated that gp120 induced the movement of lysosomes toward the cell periphery, increased lysosomal exocytosis, and enhanced release of ATP from Schwann cells, and that these processes were sensitive to P2X4 and VNUT inhibition. Using a simplified model of Schwann cell-DRG neuron interaction, we further showed that gp120-induced lysosomal exocytosis in Schwann cells increased cytosolic calcium and generation of reactive oxygen species (ROS) in DRG neurons. Our finding suggests that gp120-induced lysosome exocytotic release of ATP from Schwann cells contributes to the pathogenesis of HIV DSP.

## Materials and Methods

### Cell Culture

Primary human schwann cells (hSCs) were obtained from ScienCell (Carlsbad, CA, United States). The hSCs were cultured in Schwann Cell Medium supplemented with Schwann Cell Growth Supplement, 10% Fetal Bovine Serum and 1% Pen/Strep, and maintained at 37°C in a 5% CO_2_ atmosphere following manufacturer’s instructions. For the present study, only cells from passage 2–5 were used.

Rat Schwannoma RT4-D6P2T cells were obtained from ATCC (Manassas, VA, United States) and maintained in DMEM supplemented with 10% FBS. For the present study, only cells from passage 2–5 were used.

Rat embryonic DRG neurons were obtained from Lonza (Walkersville, MD, United States), and cultured according to manufacturer’s instructions. Rat DRG neurons were cultured in primary neuron basal medium (PNBM) supplemented with 2 mmol/L L-Glutamine, 50 μg/ml Gentamicin/37 ng/ml Amphotericin, and 2% NSF-1. To get a pure DRG neuron culture without Schwann cells, mitotic inhibitors (17.5 μg/ml uridine and 7.5 μg/ml of 5-fluoro-2′-deoxyuridine) were added. DRG neurons were used for assay between DIV 7–10.

### Plasmids and Transfection

The P2X4-pHluorin123 plasmid was a kind gift from Baljit Khakh (Addgene plasmid # 52926). The construct is based on the mouse P2X4 sequence, with the initiating methionine of pHluorin at position 123 of the reconstructed P2X4 receptor (Xu et al., 2014). The extracellular domain of P2X4 was chosen for pHluorin insertion because it avoided regions of known function. The total size of the insert was 1953 bp and was cloned into pcDNA3.1. For transient expression, RT4 Schwann cells were plated onto poly D-lysine coated 6-well plates at a density of 7000 cells/cm^2^ and transfected with 1.0 μg plasmid and 3.0 μL Lipofectamine 2000 transfection reagent at ∼70% confluency. On reaching confluency, cells were then split on to 35 mm^2^ poly D-lysine coated glass bottom dishes (MatTek) and imaged after 48–72 h.

### Antibodies and Reagents

The following primary antibodies were used in immunofluorescent staining; rabbit anti-LAMP1-CD107a (1:100 for surface staining, AB2971, EMD Millipore), rabbit anti-LAMP1-D2D11 (1:250 for intracellular staining, Catalog #9091, Cell Signaling), mouse monoclonal anti-pan cadherin (1:500, Catalog # C1821, Sigma-Aldrich), rabbit anti-TFEB (1:250, Catalog # AV100809, Sigma-Aldrich), goat anti-VNUT (1:200, Catalog # ABN110, EMD Millipore), rabbit anti-P2X4 (1:500, Catalog # AB5226, EMD Millipore), rabbit anti-RILP (1:250, Catalog # ab128616, Abcam). Alexa Fluor 594 goat anti-rabbit, 488 goat anti-rabbit, 594 goat anti-mouse, 488 goat anti-mouse antibodies were from Thermo Fisher, and Donkey Anti-Goat IgG Alexa 488 was from Abcam. All secondary antibodies were used at 1:250 dilutions.

Recombinant HIV-1 MN gp120 (Baculovirus) was from ImmunoDX (Catalog # 1021), and heat -inactivated gp120 was used as a control. Acid Phosphatase Activity Fluorometric Kit was from Sigma-Aldrich (Catalog # MAK087).

PKH 26 Red Fluorescent Cell Linker Kit was from Sigma-Aldrich (Catalog # PKH26GL). Magic Red Cathepsin B kit was from Bio-Rad (Catalog # ICT937). Lysotracker DND-99 Red (Catalog # L7528), Thermo Fisher) was used at a final concentration of 50 nmol/L, Alexa Fluor 647 Dextran (Catalog # D22914, Thermo Fisher) was used at a final concentration of 10 μg/ml, BODIPY FL ATP (Catalog # A12410, Thermo Fisher) was used at 5 μmol/L, Fluo-8 AM (Catalog # ab142773, Abcam) was used at 2 μmol/L, and H2DCFDA (Catalog # D399, Thermo Fisher) was used at 5 μmol/L.

Bx430 was from Tocris Bioscience (Catalog #5545), ATP disodium salt was from Sigma-Aldrich (Catalog# FLAAS-5VL), and clodronate disodium salt and BzATP triethylammonium salt were from Calbiochem (Catalog # 233183 and 5057340001, respectively).

### Acid Phosphatase Activity Assay

Human schwann cells were plated on to poly-D-lysine coated 24-well plates at a density of 10,000 per cm^2^ and treated with either recombinant gp120 or heat-inactivated gp120 (as control) for 24 h or 40 min. The media supernatant (500 μL) was collected and 110 μL were used per sample (in triplicates) for acid phosphatase activity assay following manufacturer’s instructions. Acid phosphatase activity was expressed as relative fluorescence, which was measured at Ex/Em-360/440 nm in a Spectra Max Plate Reader (Molecular Devices, San Jose, CA, United States).

### LAMP1 Surface Staining

Human schwann cells were plated on to poly-D-lysine coated coverslips in 24-well plates at a density of 5000 per cm^2^ and treated with either recombinant gp120 or heat-inactivated gp120 (as control) for 40 min. The plate was chilled on ice and washed twice with ice-cold PBS (containing 1 mM CaCl_2_, 2 mM MgCl_2_) and LAMP1 antibody CD107a was added in 0.1% BSA in PBS for 30 min on ice. Following three washes with ice-cold PBS, plasma membranes were co-stained with PKH26 (4 μL/mL) for 90 s on ice. The cells were then washed three times with PBS, fixed in 4% PFA for 3 min on ice, and stained with secondary antibody (0.1% BSA in PBS) for 30 min. Coverslips were then washed, and mounted on frosted glass slides (Fisher Scientific) with ProLong Gold antifade (Catalog # P36935, Thermo Fisher) before imaging on a Zeiss LSM800 Confocal Microscope.

### Surface Biotinylation Assay

Human schwann cells grown on poly-D-lysine coated 100 mm^2^ dishes were washed twice with PBS (Ca^+2^, Mg^+2^ free) and treated with recombinant gp120 (8.3 nM) or heat-inactivated gp120 (control) for 40 min. Following the treatments, cells were washed twice with ice-cold PBS and incubated with sulfo-NHS-SS-Biotin (0.5 mg/ml, 4°C, 30 min) and washed with quenching buffer (20 mM Tris and 120 mM NaCl, pH = 7.4) three-times to remove unreacted biotin. After washing twice with ice-cold PBS, hSCs were lysed with 200 μL of radioimmunoprecipitation assay (RIPA) buffer containing protease inhibitor cocktail (Catalog # A32963, Pierce, Thermo Fisher) followed by sonication. After centrifugation (10,000 × *g* for 10 min), supernatants were collected, and protein concentrations were determined with a DC protein assay (Bio-Rad). Further steps were followed as per Pierce Cell Surface Protein Isolation Kit (Catalog # 89881, Thermo Fisher). Labeled proteins were isolated with NeutrAvidin Agarose columns and eluted with sample buffer before immunoblotting. LAMP1 levels were normalized to pan-Cadherin levels for analysis.

### Immunoblotting

Cells were harvested and lysed in 1 × RIPA lysis buffer (Thermo Fisher) plus 10 mM NaF, 1 mM Na_3_VO_4_, and Protease Inhibitor Cocktail (Pierce). After centrifugation (13,000 × *g* for 10 min at 4°C), supernatants were collected, and protein concentrations were determined with a DC protein assay (Bio-Rad). Proteins (10 μg) were separated by SDS-PAGE (12% gel) and transferred to polyvinylidene difluoride membranes (Millipore). The membranes were incubated overnight at 4°C with appropriate primary and secondary antibodies. The blots were developed with enhanced chemiluminescence, and bands were visualized and analyzed by LI-COR Odyssey Fc Imaging System. Pan-Cadherin or GAPDH was used as loading control to normalize LAMP1/P2X4 and TFEB levels, respectively. Quantification of results was performed by densitometry and the results were analyzed as total integrated densitometric volume values (arbitrary units).

### ATP Measurement

ATP released from hSCs into extracellular media was measured with ATP Bioluminescent Assay Kit (Catalog # FLAA, Sigma-Aldrich) following manufacturer’s instructions. ATP levels were expressed as relative luminescence, which was measured using a Spectra Max Gemini EM plate reader (Molecular Devices).

### Immunofluorescence

Human schwann cells seeded onto poly-D-lysine coated coverslips were treated with gp120 or heat-inactivated gp120 (control) and washed twice with PBS, and fixed with 4% PFA (Electron Microscopy Sciences) for 30 min at RT. The cells were then permeabilized with 0.01% Tween-20, washed twice with PBS and blocked with 3% BSA + 1% normal goat serum for 90 min at 4°C. Incubations with primary antibodies were done overnight at 4°C followed by two washes with PBS and incubation with secondary antibody for 90 min at 4°C. Coverslips were then washed and mounted on frosted glass slides (Fisher Scientific) with ProLong Gold antifade with DAPI before imaging on a Zeiss LSM800 confocal microscope. Controls for immunostaining specificity included staining neurons with primary antibodies without fluorescence-conjugated secondary antibodies (background controls), and staining neurons with only secondary antibodies; these controls helped eliminate auto-fluorescence in each channel and bleed-through (crossover) between channels. For live cell imaging, hSCs on poly D-lysine coated 35 mm^2^ glass bottom petridishes (MatTek, P35GC-0-10-C) were transduced with BacMam Lysosome GFP for 36 h prior to imaging. Time lapse imaging with z-stacks at 2 min intervals were captured using a Zeiss LSM800 confocal microscope. Images were processed using ZEISS ZEN and Imaris 9.2 software.

### Cathepsin B Magic Red Assay

Human schwann cells were transduced with BacMAM cytosolic GFP (Thermo Fisher) for 36 h and loaded with Alexa Fluor 647 dextran (10 kDa) for 6 h, followed by a chase of 3 h. Cells were then stained with Cathepsin B Magic Red (Bio-rad) as per manufacturer’s instructions before imaging. 25–30 cells were imaged per treatment, and experiments were repeated in triplicate. Images were analyzed in Imaris with the cell and peripheral lysosome constructed as mentioned. The number of peripheral lysosomes colocalizing with Cathepsin B were quantified and expressed as a percentage relative to controls.

### BODIPY FL-ATP Staining

Human schwann cells on coverslips were transduced with BacMam lysosome RFP for 36 h, then treated and stained with BODIPY FL-ATP in media (5 μmol/L) for 30 min at 37°C, washed twice with PBS and imaged. The lysosome RFP-positive vesicles were analyzed by Imaris and classified as peripheral, juxtanuclear, or perinuclear lysosomes. Their percentages corresponding to control were then plotted in GraphPad Prism 6.0.

### DRG Neuron-Schwann Cell Conditioned Media Assay

Rat Schwannoma cells (RT4-D6P2T) grown on poly-D-lysine coated coverslips were treated with gp120 in the presence/absence of inhibitors or controls for 30 min. Of 500 μL of the extracellular media, 200 μL was used for acid phosphatase or ATP assays to confirm lysosomal exocytosis. 100 μL of the conditioned media was added to 100 μL of Fluo-8/H2DCFDA stained primary rat DRG neurons (day 10 in culture), thus having a 1:1 ratio of conditioned media:DRG neuron media. Intracellular calcium and cytosolic ROS in DRG neurons were measured with a Zeiss LSM 800 confocal microscope.

### Calcium Measurement

For Fluo-8 calcium imaging, DRG neurons were stained with Fluo-8 (2 μmol/L for 30 min at 37°C) in 1× assay buffer (HHBS with Pluronic F127 plus), followed by two washes with PBS and re-suspension in 100 μL of media prior to being taken for Schwann cell conditioned media assay. Imaging was done at Ex/Em - 490/525 nm with a 63X oil immersion lens. 25–30 cells were imaged per treatment, and the experiments were repeated in triplicate. Images were analyzed in Image J with the mean fluorescence intensity of cells (chosen as ROI) expressed as percentage. Control values were set at 100.

### ROS Measurement

Cytosolic ROS was measured by staining DRG neurons with H_2_DCFDA (5 μmol/L for 30 min at 37°C), followed by two washes and re-suspension in 100 μL of media before being taken for Schwann cell conditioned media assay. Imaging was done in the FITC channel. 25–30 cells were imaged per treatment, and the experiments were repeated in triplicate. Images were analyzed in Image J with the mean fluorescence intensity of cells (chosen as ROI) expressed as percentage. Control values were set at 100.

### LAMP1-Positive Vesicle Positioning

Confocal microscopy acquired z-stack images were analyzed and processed in Imaris 9.2 (Bitplane, Zurich, Switzerland). LAMP1/RILP/BODIPY FL-ATP-positive vesicles were processed as spots, and the nucleus as surface using the Imaris for Cell Biologists module. The distance transformation in surface function was used to create inward facing concentric surfaces from the plasma membrane, and Imaris XT was used to calculate the number of spots. All image acquisition and analytical parameters (size, thresholding) were kept the same between experiments. Particle based colocalization was carried out for all experiments.

### Statistical Analysis

All data were expressed as means ± SD. Statistical significance between two groups was analyzed with a Student’s *t-*test, and statistical significance among multiple groups was analyzed with one-way ANOVA plus a Tukey *post hoc* test. *p* < 0.05 was considered to be statistically significant.

## Results

### HIV-1 gp120 Induces Lysosomal Exocytosis

**FIGURE 1:**
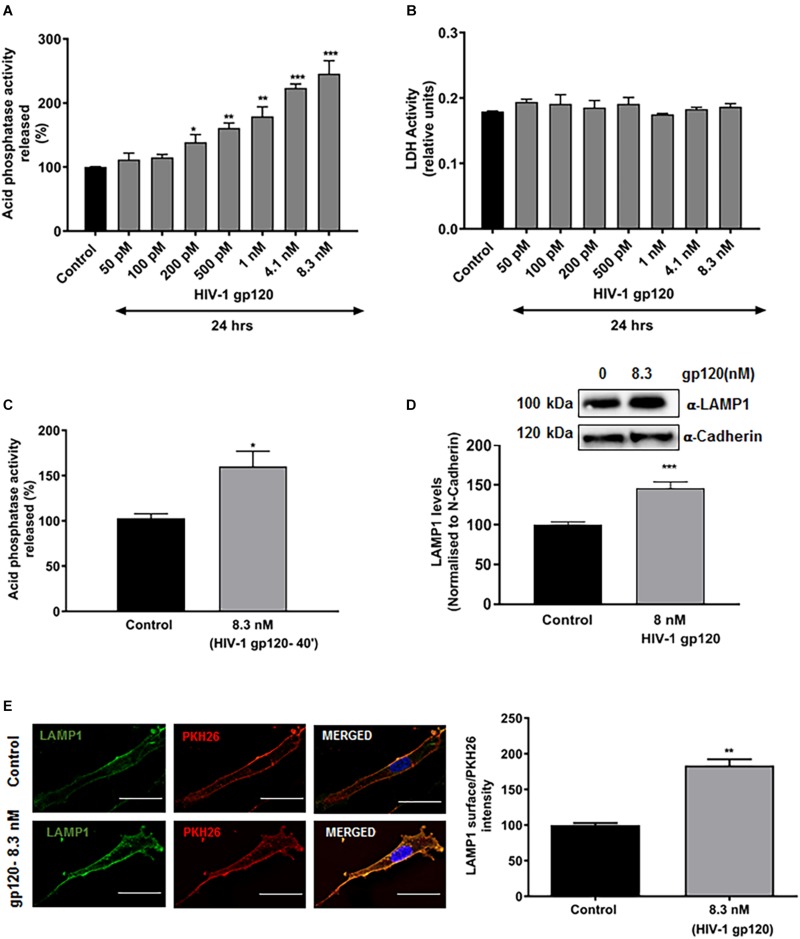
HIV-1 gp120 induces lysosomal exocytosis in primary human Schwann cells (hSCs). **(A)** Compared to heat inactivated gp120 (control), gp120 caused a concentration-dependent increase in the activity of acid phosphatase in media of hSCs (*n* = 5, ^*^p < 0.05, ^∗∗^*p* < 0.01, ^∗∗∗^*p* < 0.001). **(B)** HIV-1 gp120 did not change release of LDH in hSCs. **(C)** gp120 treatment (8.3 nmol/L for 40 min) significantly increased the release of acid phosphatase in the media of hSCs (*n* = 3, ^*^p < 0.05). **(D)** In surface protein biotinylation assay, gp120 treatment (8.3 nmol/L for 40 min) increased PM levels of LAMP1 (*n* = 3, ^∗∗∗^*p* < 0.001). **(E)** In surface protein labeling assay, gp120 treatment (8.3 nmol/L for 40 min) increased PM of LAMP1 (*n* = 3, ^∗∗^*p* < 0.01; bar = 15 μm).

Characteristic hallmarks of lysosomal exocytosis are increased release of lysosomal hydrolases into extracellular media and the translocation of lysosomal membrane proteins to the plasma membrane (PM) (Zhang et al., 2007; Medina et al., 2011). Using these hallmarks, we determined the extent to which gp120 affects lysosomal exocytosis in primary hSCs. Release of lysosomal hydrolases into extracellular media was quantified by measuring activity of acid phosphatase in the media. Incubation of hSCs with recombinant HIV-1 gp120 for 24 h resulted in a concentration-dependent increase in release of lysosomal acid phosphatase into media (Figure 1A). Under these conditions, gp120 treatment did not induce significant cell death, as indicated by no changes in levels of released LDH (Figure 1B). A shorter treatment for 40 min with 8.3 nmol/L of HIV-1 gp120 also led to significant increase in acid phosphatase release into media (Figure 1C). This shorter treatment was therefore used for all subsequent experiments. The PM translocation of LAMP1 was assessed by surface biotinylation assay, and PM levels of LAMP1 were normalized to a control PM protein (α-cadherin). We demonstrated that gp120 (8.3 nmol/L) treatment for 40 min significantly increased PM protein levels of LAMP1 (Figure 1D). Furthermore, the PM translocation of LAMP1 was also assessed with surface staining of LAMP1, and levels of surface expression of LAMP1 was calculated as the ratio of LAMP1 immunofluorescent signal to total PM fluorescent signal as labeled with PKH26. gp120 (8.3 nmol/L) treatment for 40 min significantly increased surface expression of LAMP1 (Figure 1E). Together, these findings indicate that gp120 induces lysosomal exocytosis in hSCs.

### HIV-1 gp120 Induces Redistribution of Lysosomes

**FIGURE 2:**
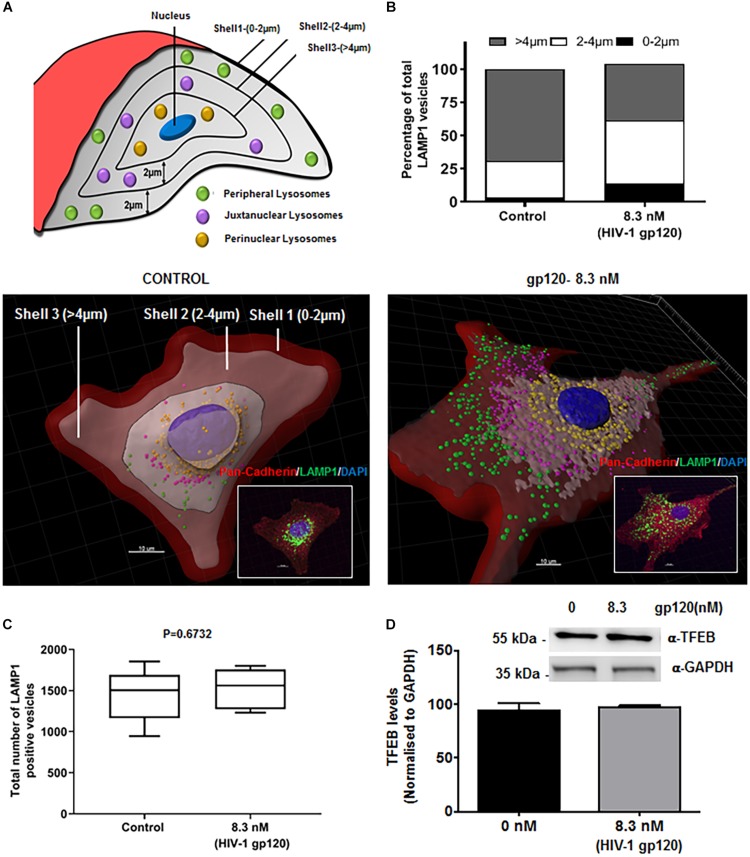
HIV-1 gp120 causes lysosomal redistribution in hSCs. **(A)** Classification scheme of LAMP1 positive lysosomes in concentric shells labeled as peripheral (0–2 μm from PM), juxtanuclear (2–4 μm from PM), and perinuclear (>4 μm from PM) lysosomes. **(B)** As shown in representative LAMP1 staining (insert) and 3D reconstructed images, gp120 treatment (8.3 nmol/L for 40 min) increased the percentage of peripheral (green) and juxtanuclear (magenta) lysosomes and decreased the percentage of perinuclear (yellow) lysosomes. Plasma membranes were outlined with Pan-Cadherin (red) and the nucleus stained with DAPI (blue). **(C)** gp120 treatment (8.3 nmol/L for 40 min) did not change total numbers of LAMP1 positive lysosomes (*n* = 100) **(D)** gp120 treatment (8.3 nmol/L for 40 min) did not change TFEB protein levels in hSCs.

Lysosome exocytosis requires two sequential steps. First, lysosomes are recruited to the close proximity of the cell surface (docking), and second, the pool of pre-docked lysosomes then fuses with the PM and releases lysosomal contents (Andrews, 2000; Martinez et al., 2000). The molecular trafficking machinery involved in these two steps is only partially known. To further determine mechanisms by which gp120 affects lysosomal exocytosis, we determined the effects of gp120 on the positioning of LAMP1-positve lysosomes. hSCs were treated with gp120 (8.3 nmol/L) for 40 min and stained with LAMP1 antibodies. Z-stacks were acquired every 0.4 μm in order to approximately position each lysosome in a single z plane. These images were then reconstructed in three dimensions with Imaris software; the cell boundary was either labeled by pan-cadherin antibodies or drawn from DIC images. As illustrated in Figure 2A concentric shells of 2 μm diameter were constructed, moving radially inward from the PM toward the nucleus, and labeled as Shell 1, 2, and 3. The lysosomes in Shell 1, 2, and 3 were labeled as peripheral, juxtanuclear, and perinuclear, respectively. As shown in Figure 2B, gp120 treatment increased the percentage of both peripheral and juxtanuclear lysosomes and decreased the percentage of perinuclear lysosomes. These findings indicate that gp120 causes a redistribution of LAMP1-positive lysosomes; lysosomes moving from the perinuclear to peripheral positions as a result of gp120 treatment. Given that these static images might not capture the dynamics of lysosomal exocytosis, we tracked the movement of LAMP1-positive lysosomes using live cell imaging. In hSCs transduced with LAMP1-GFP, gp120 promoted lysosomes trafficking to the PM, as indicated by increased number of LAMP1-positive lysosomes in the outermost Shell 1 (Supplementary Figure S1A). To rule out the possibility that the percentage changes of LAMP1-positive lysosomes in three shells is not a result of changes in lysosome biogenesis, total numbers of LAMP1-positive vesicles were quantified by immunostaining with LAMP1 antibodies. Treatment with gp120 (8.3 nmol/L) for 40 min did not change total number of LAMP1 positive vesicles (Figure 2C). Treatment with gp120 (8.3 nmol/L) for 40 min did change total protein levels of TFEB, a key transcription factor that regulates the biogenesis of lysosome and autophagy (Figure 2D). Furthermore, nuclear translocation of TFEB was determined with immunostaining, and gp120 (8.3 nmol/L) treatment for 40 did not induce significant change in nuclear translocation of TFEB (Supplementary Figure S1B). Together, these results indicate that gp120 induces lysosomal exocytosis in hSCs. Thus, our findings suggest that gp120 enhances the trafficking of lysosomes toward cell surfaces and promotes lysosomal exocytosis.

### HIV-1 gp120-Induced Redistribution of Lysosomes Display Functional Heterogeneity

**FIGURE 3:**
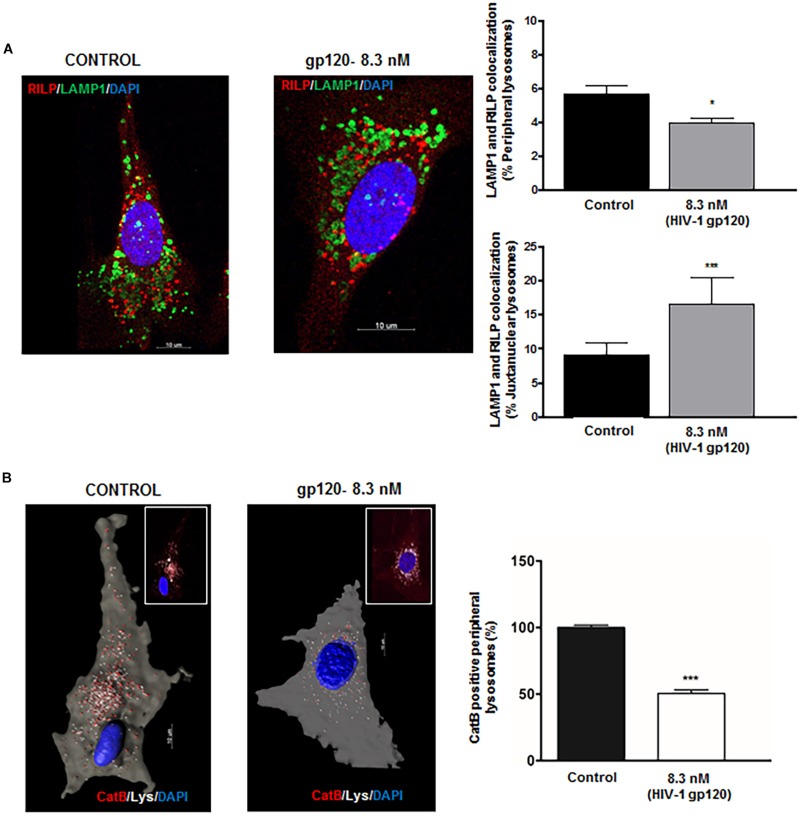
HIV-1 gp120-induces redistribution of lysosomes displays functional heterogeneity. **(A)** gp120 treatment (8.3 nmol/L for 40 min) significantly decreased colocalization of LAMP1 (green) with RILP (red) in peripheral lysosomes, but increased colocalization of LAMP1 with RILP in juxtanuclear lysosomes (*n* = 3, ^*^p < 0.05, ^∗∗∗^*p* < 0.001). **(B)** As shown in representative confocal (inset) and reconstructed Imaris images, gp120 treatment (8.3 nmol/L for 40 min) significantly reduced Cathepsin B activity (red) in peripheral (white) lysosomes (*n* = 3, ^∗∗∗^*p* < 0.001).

A recent study has shown that perinuclear lysosomes are more acidic and have higher cathepsin L activity than peripheral lysosomes (Johnson et al., 2016). This heterogeneity could be due to the stabilization of the v-ATPase subunit V1G1 by rab interacting lysosomal protein (RILP), a downstream effector of Rab7 that links Rab7 to dynein–dynactin and controls retrograde transport of late endosomes and lysosomes (Johansson et al., 2007). To further understand the underlying mechanism by which gp120 affects lysosome trafficking and lysosomal exocytosis, we determined the effects of gp120 on the colocalization of LAMP1 with RILP in hSCs. We found that gp120 (8.3 nmol/L) treatment for 40 min significantly lowered the association of RILP with peripheral lysosomes upon gp120 treatment but increased the association for juxtanuclear lysosomes (Figure 3A). These findings are consistent with our observations that gp120 induced the movement of lysosomes from the juxtanuclear to the peripheral position and enhanced lysosomal exocytosis. Following upon others’ observations that peripheral lysosomes are de-acidified compared to juxtanuclear and perinuclear lysosomes (Johnson et al., 2016), we assessed pH-dependent lysosome degradative capacity using a Magic Red Cathepsin B assay. This assay utilizes a fluorogenic substrate of the lysosomal hydrolase Cathepsin B, which is active under acidic pH, and loses activity upon de-acidification. The amount of fluorescence is therefore a direct output of active Cathepsin B, indicating the acidification status of the lysosome (Linke et al., 2002; Cheng et al., 2018). For this assay, hSCs were transfected with cytosolic GFP, and pulsed with Alexa Fluor 647 dextran (10 kDa) for 6 h followed by a chase of 3 h in order for the dextran to travel via the endocytic pathway to lysosomes. The hSCs were then treated with gp120 (8.3 nmol/L) for 40 min, stained with Magic Red Cathepsin B and imaged live by confocal microscopy. As expected, peripheral lysosomes had significantly lower levels of Magic Red fluorescence, indicating decreased activity of cathepsin B (Figure 3B). These findings indicate that the altered distribution of lysosomes upon gp120 treatment could be attributed to lysosome de-acidification. To further test the extent to which reduced v-ATPase activity and lysosome de-acidification promotes lysosome exocytosis, we treated cells with bafilomycin, a specific v-ATPase inhibitor, and determined the distribution of LAMP1-positive lysosomes. LAMP1 positive lysosomes were analyzed for their distance from the PM (cells outlined by Pan-Cadherin labeling) using the same methods as mentioned earlier in Figures 2C,D. Similar to that of gp120, treatment with bafilomycin increased the percentage of both peripheral and juxtanuclear lysosomes and decreased the percentage of perinuclear lysosomes (Supplementary Figure S2).

### P2X4 Is Involved in gp120-Induced Lysosomal Exocytosis

**FIGURE 4:**
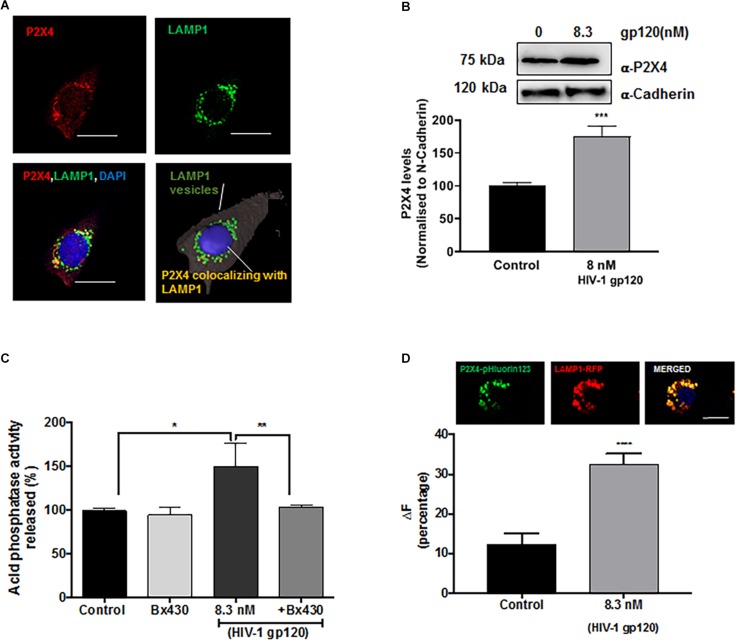
Lysosomal P2X4 channel is involved in HIV-1 gp120 induced lysosomal exocytosis. **(A)** Representative confocal and reconstructed Imaris images show P2X4 (red) is colocalized with LAMP1 (green) positive lysosomes in hSCs (bar = 20 μm). **(B)** In surface biotinylation assay, gp120 treatment (8.3 nmol/L for 40 min) increased P2X4 translocation to the PM in hSCs (*n* = 3, ^∗∗∗^*p* < 0.001). **(C)** An allosteric P2X4 regulator Bx430 (0.5 μmol/L) prevented gp120 (8.3 nmol/L for 40 min)-induced increases in the activity of acid phosphatase release in media of hSCs (*n* = 3, ^*^p < 0.05, ^∗∗^*p* < 0.01). **(D)** As shown in RT4 rat Schwann cells co-transfection of P2X4-pHluorin123 (GFP) and LAMP1-RFP (bar = 10 μm), gp120 treatment (8.3 nmol/L for 40 min) increased fluorescence of P2X4-pHluorin123 (*n* = 3, ^∗∗∗^*p* < 0.001).

Although lysosomes are recruited to being in close proximity to the cell surface in a Ca^2+^-independent manner (Rodríguez et al., 1997; Andrews, 2000; Jaiswal et al., 2002), the fusion of pre-docked lysosomes with the PM requires Ca^2+^ elevation. Mounting evidence indicate that calcium released from lysosomes could mediate such a fusion event, and recently studies have shown that P2X4 cationic channels are localized to both plasma membranes and lysosomes and is regulated by its natural ligand ATP in a pH-dependent manner (Cao et al., 2015; Dong, 2015). Thus, gp120-induced lysosomal exocytosis could result from activation of P2X4 cationic channels. To determine the involvement of P2X4 channel in gp120-induced lysosomal exocytosis, we first characterized the subcellular localization of P2X4 in hSCs. We demonstrated that P2X4 channels colocalized with LAMP1-positive lysosomes in hSCs (Figure 4A). Using a surface biotinylation assay, we demonstrated that gp120 (8.3 nmol/L) treatment for 40 min increased PM levels of P2X4 (Figure 4B), indicating the involvement of P2X4 in gp120-induced lysosome exocytosis. To further confirm the role of P2X4 in gp120-induced lysosomal exocytosis, pre-incubation with Bx430, a selective allosteric antagonist of P2X4 (Ase et al., 2015), significantly blocked gp120-induced increases in release of lysosomal acid phosphatase (Figure 4C). We further determined the effect of a P2X4 agonist 2,3′-O-(4-benzoyl)benzoyl-ATP (BzATP) on lysosomal exocytosis (Emmett et al., 2008; Stokes et al., 2011). Similar to gp120, BzATP (5.0 μmol/L for 30 min) promoted lysosome exocytosis as indicated by increased activity of acid phosphatase released in media (Supplementary Figure S3). Thus, gp120 could induce lysosomal exocytosis by activating lysosomal P2X4 channels, and it is possible that gp120 affects the activity of P2X4 within the lumen of lysosomes because gp120 can be endocytosed (Berth et al., 2015; Wenzel et al., 2017). In support, we demonstrated that significant amounts of exogenous gp120-FITC trafficked to lysosomes in as early as 30 min with a Pearson’s coefficient of 0.456 (Supplementary Figure S4B). It is known that the acidic pH of lysosome lumen has been shown to inhibit P2X4 channels (Huang et al., 2014). Therefore, upon gp120 treatment, lysosomes might move to the periphery, their luminal pH might increase (de-acidification), and as a result P2X4 receptors might be activated. To test this hypothesis, rat Schwannoma cells (RT4-D6P2T) were co-transfected with P2X4-tagged with pH sensitive fluorescent pHluorin123 and LAMP1-RFP. The pHluorin is a pH-sensitive GFP variant, the fluorescence of which remains quenched at acidic pH and increases as the pH increases to neutral (Xu et al., 2014). Cells co-transfected with P2X4-pHluorin123 and LAMP1-RFP were treated with gp120 (8.3 nmol/L) and imaged by time lapse microscopy for 20 min. As expected, gp120 increased P2X4-pHluorin123 fluorescence (Figure 4D), indicating gp120 de-acidifies P2X4 positive lysosomes. To confirm that RT4 rat Schwann cells behave the same as that of primary hSCs in response to gp120 treatment, we determined the effect of gp120 on lysosome exocytosis in RT4 rat Schwann cells. We found that the response of these rat Schwann cells to gp120 was similar to that of primary hScs; gp120 increased the release of lysosomal acid phosphatase in media of rat Schwann cells (Supplementary Figure S4A).

### HIV-1 gp120 Modulates Lysosomal P2X4 via VNUT ATP Transporter

**FIGURE 5:**
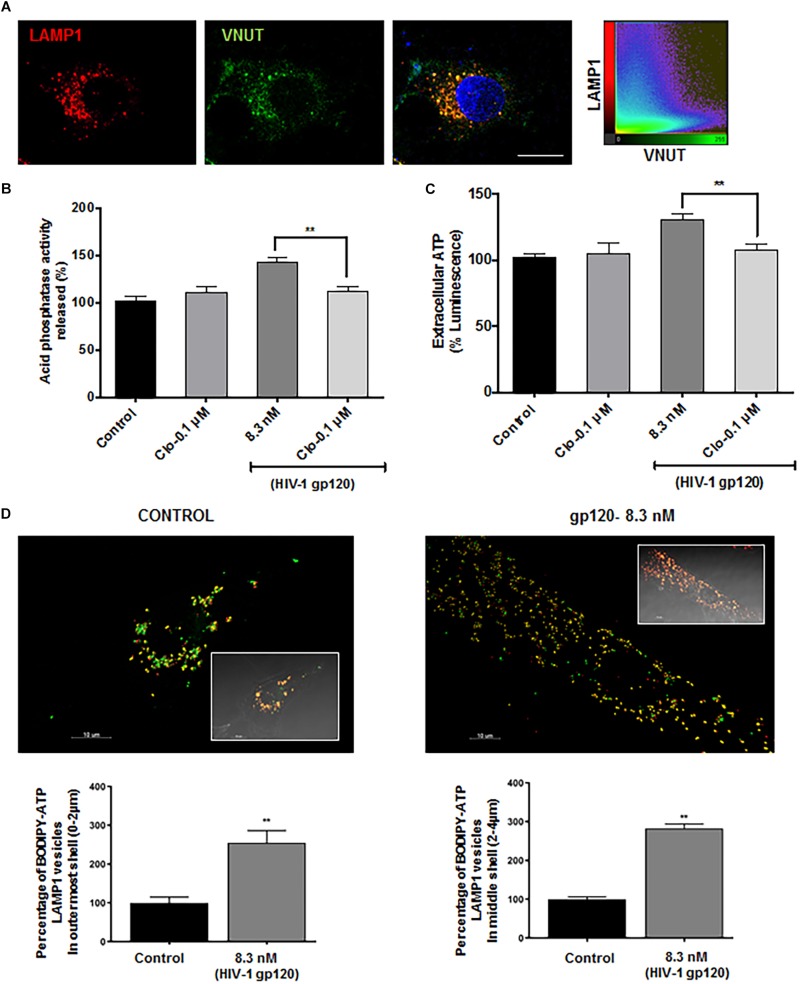
Lysosomal ATP transporter-VNUT mediates HIV-1 gp120 induced lysosomal exocytosis. **(A)** Representative confocal images and scatterplot show the colocalization of VNUT (green) with LAMP1 (red) positive lysosomes in hSCs (bar = 15μm). **(B)** Inhibiting VNUT with clodronate (Clo, 0.1 μmol/L) prevented gp120 (8.3 nmol/L for 40 min)-induced increases in the activity of acid phosphatase release in media of hSCs (*n* = 3, ^∗∗^*p* < 0.01). **(C)** gp120 treatment (8.3 nmol/L for 40 min) increased ATP levels in media of hSCs, and this effect was prevented by inhibiting VNUT with clodronate (Clo, 0.1 μmol/L) (*n* = 3, ^∗∗^*p* < 0.01. **(D)** As shown in representative Imaris reconstructed and confocal images (inset), gp120 (8.3 nmol/L for 40 min) increased percentage of ATP (BODIPY FL-ATP, green) in peripheral and juxtanuclear lysosomes (LAMP1-RFP) (*n* = 3, ^∗∗^*p* < 0.01).

Lysosomal P2X4 activity has been shown to be dually regulated by luminal pH and ATP. One of the key lysosomal ATP transporters identified is VNUT (Vesicular Nucleotide Transporter or SLC17A9), responsible for ATP transport into lysosomes. VNUT has been shown to play a role in lysosomal exocytosis leading to extracellular release of ATP. To determine the involvement of VNUT in gp120-induced lysosome exocytosis, the lysosomal localization of VNUT in hSCs was first confirmed by immunocytochemistry, showing that 85% of LAMP1-positive lysosomes co-localized with VNUT, measured using mander’s colocalization coefficient (MCC) in Imaris software (Figure 5A). Importantly, pretreatment with clodronate, a specific allosteric inhibitor of VNUT, blocked gp120-mediated acid phosphatase release (Figure 5B) as well as ATP release (Figure 5C) into the extracellular media (Kato et al., 2017). To further determine the involvement of lysosomal ATP in gp120-induced lysosomal exocytosis, we visualized directly lysosomal ATP and their redistribution upon gp120 treatment. Here, hSCs were transduced with LAMP1-RFP, labeled with BODIPY FL-ATP and imaged upon gp120 treatment. The majority of BODIPY FL-ATP staining was observed to be in the lysosomes. To determine the redistribution of lysosomal ATP, cell boundaries were drawn from DIC images and vesicles positive for both LAMP1 and BODIPY FL-ATP were classified based on their distance from the PM as mentioned earlier. As shown in Figure 5D, gp120 treatment resulted in a significant increase in the percentage of ATP positive lysosome in peripheral and juxtanuclear regions. Combined, these results suggest that lysosome de-acidification and ATP-mediated P2X4 activation could be a key process in gp120-induced lysosomal exocytosis.

### HIV-1 gp120-Induced Schwann Cell Lysosomal Exocytosis Affects DRG Neuron Physiology

**FIGURE 6:**
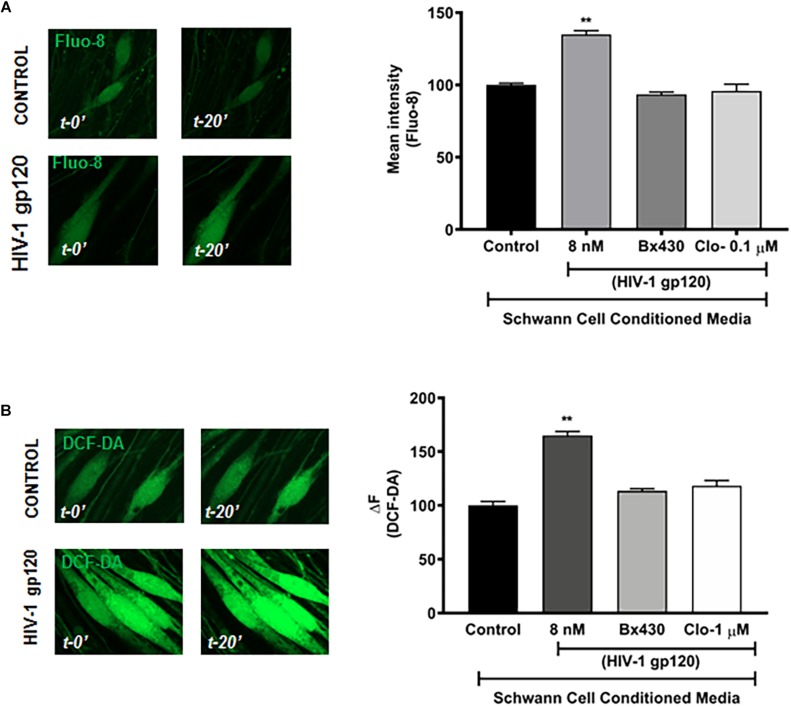
HIV-1 gp120 conditioned RT4 Rat Schwann cell media increases cellular calcium and ROS in DRG neurons. **(A)** gp120 conditioned Schwann cell media, but not gp120 + Bx430 or gp120 + Clo conditioned media, increased intracellular calcium levels as measured by Fluo-8 in rat DRG neurons (*n* = 3, ^∗∗^*p* < 0.01). **(B)** gp120 conditioned media, but not gp120 + Bx430 or gp120 + Clo conditioned media, increased cytosolic ROS levels as measured by DCF-DA in rat DRG neurons (*n* = 3, ^∗∗^*p* < 0.01).

To further determine whether gp120-induced lysosomal exocytosis in Schwann cells could affect the function of DRG neurons and contribute to HIV neuropathy, conditioned media of Schwann cells was used for treatment of DRG neurons. Here, RT4 rat Schwann cells were treated with HIV-1 gp120 for 45 min in the presence or absence of P2X4 blocker (Bx430) and VNUT inhibitor (clodronate), and the extracellular media was added to primary rat DRG neuronal cultures in a 1:1 ratio. Given that a rise in intracellular calcium and increasing the excitability of DRG neurons has been linked to heightened nociceptive sensation in neuropathic pain (Kostyuk et al., 2001; Chung and Chung, 2002; Yang et al., 2018), we measured intracellular calcium of DRG neurons with Fluo-8. We found that gp120-treated Schwann cell conditioned media elevated intracellular Ca^2+^ in DRG neurons, whereas conditioned media from P2X4 and VNUT inhibition (by Bx430 and clodronate, respectively) failed to induce a rise in intracellular calcium (Figure 6A). These findings indicate that gp120-induced lysosomal exocytosis of ATP in hSCs could be a key factor causing intracellular calcium to rise in DRG neurons. To further assess DRG neuronal function, cytosolic ROS was measured by staining with DCF-DA. As shown in Figure 6B, incubation with gp120-treated Schwann cell conditioned media significantly increased DRG neuron ROS levels, whereas conditioned media from P2X4 and VNUT inhibition (by Bx430 and clodronate, respectively) failed to induce a rise in ROS levels. Because lysosomes have been shown to store ATP, which can be released upon exocytosis in other cell types (Zhang et al., 2007; Sivaramakrishnan et al., 2012; Beckel et al., 2018) and we found that gp120 increased the release of ATP into media (Figure 5), we determined effects of exogenous ATP on intracellular Ca^2+^ and ROS in DRG neurons. We found that ATP treatment increased both cytosolic Ca^2+^ and ROS in DRG neurons in a concentration-dependent manner (Supplementary Figures S5A,B).

## Discussion

HIV-associated distal symmetric polyneuropathy (DSP) is the most common neurological complication of HIV infection that results in chronic debilitating neuropathic pain. There is an urgent need for better understanding of its pathogenesis and for the development of effective therapeutic strategies. In the present study, we demonstrated that HIV-1 gp120 promoted the movement of lysosomes toward plasma membranes followed subsequently by lysosomal exocytosis and release of ATP; this process is achieved by gp120-induced activation of lysosome P2X4 via lysosome de-acidification and VNUT coupling. Such gp120-induced lysosomal exocytosis and release of ATP from Schwann cells induced increases in cytosolic calcium and ROS in DRG neurons. Our results suggest that gp120-induced lysosomal exocytotic release of ATP might signal through P2X4 and VNUT in Schwann cells and thereby contribute to the pathogenesis of HIV-DSP.

The HIV-1 envelope glycoprotein gp120, which is non-covalently attached to the transmembrane gp41 protein, is expressed on the outer layer of the virus. As such, gp120 is readily shed from HIV-1 virions and infected cells (Gelderblom et al., 1987; Layne et al., 1992; Wyatt and Sodroski, 1998). Detectable levels of gp120 in plasma (0.5–15.6 ng/ml) and in spleen and lymph node (>0.3 ng/ml) is present in HIV-1 infected individuals (Oh et al., 1992; Santosuosso et al., 2009; Rychert et al., 2010). Even in HIV-infected individuals under ART with no detectable viral replication, gp120 can be detected in lymph nodes (Popovic et al., 2005). As the HIV-1 envelope protein, gp120 is critical for virus infection, because it is necessary for binding to specific cell surface receptors (CD4, CXCR4, and CCR5) on target cells and facilitating virus entry (Deng et al., 1996). Released free gp120 is a potent HIV virotoxin via either indirect mechanisms whereby gp120 promotes the release of pro inflammatory cytokines or other neurotoxic factors that elicit indirect neurotoxic effect (Kaul and Lipton, 1999; Bezzi et al., 2001), or direct mechanisms whereby gp120 elicits direct neurotoxic effect in the absence of glutamate receptor activation or pro-inflammatory cytokines (Bachis and Mocchetti, 2004; Bachis et al., 2006; Wenzel et al., 2017).

The role of gp120 in the pathogenesis of HIV-DSP has long been recognized. Higher levels of gp120 in the spinal dorsal horn of HIV neuropathic pain-positive individuals provides a clue that gp120 plays an important role in the development of HIV DSP (Yuan et al., 2014). Exposure of peripheral nerves to HIV-1 gp120 results in neuropathic pain (Herzberg and Sagen, 2001). HIV gp120 transgenic mice or rodents treated with gp120 develop neuropathic pain with pathological similarities with humans such as peripheral neuropathy, pain behavior, synaptic degeneration and activation of glial cells (Nagano et al., 1996; Oh et al., 2001; Wallace et al., 2007; Hao, 2013; Burdo and Miller, 2014). Similar to gp120-induced neurotoxicity in CNS, gp120 could elicit direct neurotoxicity on DRG neurons (Apostolski et al., 1993, 1994) gp120 could activate macrophages, which in turn release neurotoxic inflammatory mediators that lead to indirect neurotoxicity, or gp120 acts on Schwann cells to release RANTES, which leads to DRG neurotoxicity via CCR5 activation (Keswani et al., 2003; Melli et al., 2006).

Additionally, there is evidence that organelle damage, such as mitochondrial dysfunction (Lehmann et al., 2011) and endoplasmic reticulum dysfunction (Hoke et al., 2009), contributes to the development of HIV DSP. However, virtually nothing is known about the involvement of lysosomes, which not only plays an important role in intercellular communication via lysosome exocytosis in CNS (Zhang et al., 2007; Li et al., 2008; Liu et al., 2011) but also play an important role in regulating Schwann cell function such as regulating myelination and the release of ATP, an important mediator of peripheral pain (Chen et al., 2012; Shin et al., 2014; Brosius Lutz et al., 2017). As such, we determined the extent to which and mechanisms by which gp120 affects lysosomal exocytosis in Schwann cells and contributes to HIV neuropathy.

### Mechanism of gp120-Induced Lysosome Exocytosis in Schwann Cells

By measuring increased release of lysosomal hydrolases (acid phosphatase) into extracellular media and the translocation of lysosomal membrane proteins (LAMP1) to the PM, we demonstrated that gp120 promoted lysosome exocytosis in human Schwann cells. Because lysosomes docking (calcium independent) near the PM and subsequent fusion (calcium-dependent) with the PM are the two key steps to lysosome exocytosis (Andrews, 2000; Jaiswal et al., 2002; Tucker et al., 2004), we then determined the extent to which gp120 affected lysosome positioning, an important factor mediating cellular homeostasis including autophagy, apoptosis, exosome release and metabolic signaling (Korolchuk et al., 2011; Eitan et al., 2016; Jia et al., 2017). To estimate lysosomal positioning, we used published methods of classifying them as perinuclear, juxtanuclear, or peripheral lysosomes based on their distances from the PM in concentric rings (Fan and He, 2016; Johnson et al., 2016). Here, we used an increased ring diameter of 2 μm owing to the large size of hSCs, and we demonstrated that short-term treatment (40 min) with gp120 did not change total numbers of LAMP1-positive lysosomes but increased the numbers of peripheral and juxtanuclear lysosomes with concurrent decrease in the numbers of perinuclear lysosomes. Thus, gp120 increases the docking of lysosomes near the PM, which is consistent with enhanced lysosome exocytosis.

Lysosome trafficking in polarized cells occurs along microtubules and is mediated by plus-end directed kinesin (Hollenbeck and Swanson, 1990) and minus-end directed dynein motors (Harada et al., 1998) that are controlled by the action of GTPases such as Rab proteins (Progida and Bakke, 2016; Langemeyer et al., 2018). Rab7 is a specific marker for late endosomes-lysosomes (Feng et al., 1995; Meresse et al., 1995), and its downstream effector protein RILP induces recruitment of lysosomes to the dynein-dynactin complex preventing further cycling of Rab7 (Jordens et al., 2001). Here, we showed that gp120 decreased the association of peripheral lysosomes with RILP, thus further supporting the concept that gp120 promotes the trafficking of lysosomes progressing toward exocytosis (Johnson et al., 2016). We also demonstrated that gp120 increased the association of RILP with juxtanuclear lysosomes, which indicated that only the peripheral lysosomes undergo exocytosis. We further show that gp120 decreased cathepsin B activity in these peripheral lysosomes. Given that cathepsin B is more active in acidic environment, our findings are consistent with recent findings that lysosomes undergo progressive de-acidification as they move toward the PM (Johnson et al., 2016), and possibly these peripheral lysosomes have fulfilled their degradative functions and are destined for exocytosis.

Upon further investigating the underlying mechanisms whereby gp120 affects lysosomal trafficking to the cell periphery and lysosome exocytosis, we determined the involvement of P2X4, a fast and sensitive purinergic receptor that preferentially localizes to lysosomes (Xu et al., 2014) and its activity is dually regulated by intra-lysosome pH and intra-lysosome ATP (Huang et al., 2014; Cao et al., 2015). In the present study we did not focus on other purinergic receptors, such as P2X7 that have been implicated in modulating lysosomal pH (Takenouchi et al., 2009; Guha et al., 2013) because it has been shown that P2X7 receptors have ER and PM localization rather than lysosomes (Murrell-Lagnado and Robinson, 2013) owing probably to its longer C-terminal (Smart et al., 2003). We show that P2X4 is localized to LAMP1 vesicles in hSCs, an observation that is consistent findings in rat Schwann cells (Su et al., 2019). Importantly, blocking P2X4 with specific human P2X4 antagonist Bx430 attenuated gp120-induced lysosomal exocytosis. The activity of lysosomal P2X4 is normally inhibited under acidic luminal pH (Huang et al., 2014). As lysosomes undergo alkalization while moving toward the PM (Johnson et al., 2016), the de-acidification of lysosomes could activate P2X4. Using P2X4-pHluorin 123 construct, whose fluorescence increases with increasing pH, we demonstrated that gp120 treatment increased the fluorescence signal of lysosomes expressing P2X4-pHluorin 123, indicating gp120 increases lysosome pH (de-acidification).

However, increased lysosome pH itself is not sufficient to activate P2X4 as it is under the dual regulation of luminal pH and ATP. Consistent with others’ findings (Shin et al., 2012), we demonstrated that lysosomes serve as ATP rich stores. Importantly, we demonstrated that gp120 treatment increased the percentage of ATP rich peripheral lysosomes. Thus, this population of ATP rich, de-acidified peripheral lysosomes is ideal for P2X4 activation, which probably reflects the calcium dependent second step of lysosomal exocytosis. We further showed that the ATP transporter VNUT was responsible for Schwann cell lysosomal ATP filling as inhibiting VNUT prevented gp120-induced lysosomal exocytosis and ATP release in the extracellular media. Thus, consistent with a report that functional coupling of VNUT and P2X4 underlies lysosome exocytosis (Zhong et al., 2016), our findings indicate such a functional coupling of VNUT and P2X4 receptors play a role in gp120 induced lysosomal exocytosis. It is well known that lysosomal exocytosis is a calcium-dependent process; increases in intracellular calcium has been shown to promote the fusion of lysosomes within the vicinity of the PM and induce lysosomal exocytosis (Jaiswal et al., 2002). Calcium released via P2X4 receptors may also contribute to gp120-induced lysosomal exocytosis. Activation of PM P2X4 receptors allows the influx of Ca^2+^ leading to increased intracellular Ca^2+^. Apart from calcium influx across PM, intracellular Ca^2+^ can be also increased via calcium released from lysosome lumen upon lysosomal P2X4 activation (Cao et al., 2015). Thus, calcium influx across PM and calcium release from lysosomes could both contribute to HIV-1 gp120-induced lysosomal exocytosis.

Together, our findings suggest that gp120 promotes the movement of lysosomes toward plasma membranes and subsequent lysosome exocytosis, and this process is achieved by gp120-induced activation of lysosome P2X4 via lysosome de-acidification and ATP enrichment. Currently, it is not clear how gp120 leads to lysosome de-acidification and ATP enrichment in Schwann cells. Given that gp120 has been shown to bind to CXCR4 receptors on Schwann cells (Keswani et al., 2003) and that gp120 can be endocytosed and localized to lysosomes within 30 min (Supplementary Figure S4B) (Costantini et al., 2015; Wenzel et al., 2017), gp120 could affect lysosome pH, lysosome ATP transport, and activity of lysosomal P2X4 either directly at the luminal side of lysosomes or indirectly via receptor-mediated signaling. Thus, further mechanistic studies are warranted.

### The Functional Importance of gp120-Induced Lysosome Exocytosis in Schwann Cells

Schwann cells are the most abundant glial cells of the peripheral nervous system, ensheathing all axons of peripheral nerves as either myelinating or non-myelinating cells. In addition to its role as insulators of axons, Schwann cells are crucial for the proper function and maintenance of peripheral nerves by providing metabolic or trophic support (Beirowski et al., 2014; Feldman et al., 2017; Sasaki et al., 2018) and modulating responses to nerve injury (Jessen and Mirsky, 2016; Kim et al., 2018). On the other hand, the presence of axons is crucial to Schwann cells’ ability to de-differentiate (Jang et al., 2017, Soto and Monje, 2017; Norrmén et al., 2018). Thus, disrupting Schwann cell function can compromise glial–axon communication that can lead to disturbed nerve homeostasis and ultimately lead to fiber loss, neurodegeneration, and pain.

In the central nervous system, lysosomal exocytosis represents a new pathway for gliotransmitter secretion from astrocytes (Zhang et al., 2007; Li et al., 2008; Liu et al., 2011). In Schwann cells, lysosome exocytosis has also been demonstrated and plays an important role in regulating myelination (Chen et al., 2012; Shin et al., 2012; Jung et al., 2014; Su et al., 2019). In particular, release of ATP via lysosome exocytosis has been demonstrated in Schwann cells (Shin et al., 2012). Numerous studies have shown the effect of extracellular ATP on Schwann cell physiology including its role on differentiation (Stevens and Fields, 2000), activation (Rodella et al., 2017), and its role in myelination (Ino et al., 2015) and in Wallerian degeneration (Shin et al., 2012, 2014). The over-expression of P2X4 in Schwann cells have recently been shown to promote remyelination via secretion of brain derived neurotrophic factor (Su et al., 2019), and another P2X receptor P2X7 has been shown to be involved in Charcot-Marie-Tooth disorder, a common inherited human neuropathy with demyelination (Nobbio et al., 2009). On the other hand, ATP is recognized as an important mediator of peripheral pain via activating the purinergic receptors and a rise in intracellular calcium in DRG neurons (Surprenant et al., 1996; Qi et al., 2011; Hattori and Gouaux, 2012; Masuda et al., 2016; Yamashita et al., 2016; Jurga et al., 2017; Ying et al., 2017; Yang et al., 2018).

In the present study, we explored the effect of gp120-induced lysosome exocytotic release of ATP from Schwann cells on DRG neuron function by treating primary culture rat DRG neurons with conditioned media from gp120-treated rat Schwannoma cells. We demonstrated that conditioned media from gp120-treated rat Schwannoma cells induced a rise in the levels of intracellular calcium and increase in cytosolic ROS in DRG neuron, both of which have been implicated in the development of peripheral neuropathy and/or HIV DSP (Salvemini et al., 2011; Materazzi et al., 2012; Iida et al., 2016). Importantly, conditioned media from gp120-treated rat Schwannoma cells in the presence of P2X4 or VNUT antagonists failed to induce rises in calcium and ROS in DRG neurons. Given that blocking either P2X4 or VNUT in Schwann cell attenuated gp120-induced lysosome exocytosis and the release of ATP into media, our findings suggest that ATP released via lysosome exocytosis from Schwann cells induces calcium rise in DRG neuron. However, our findings could not exclude the involvement of other factors released via lysosome exocytosis from Schwann cells. For instance, Schwann cell exosomes have been shown to interact with axons, and increased miRNA455-3p induction has been shown to be associated with HIV DSP (Lopez-Verrilli and Court, 2012; Lopez-Leal and Court, 2016; Jia et al., 2018; Zhou et al., 2018).

In summary, our findings suggest that HIV-1 gp120 promotes the movement of lysosomes toward plasma membrane followed subsequently by lysosomal exocytosis and the release of ATP; and this process is achieved by gp120-induced activation of lysosome P2X4 via lysosome de-acidification and VNUT coupling. Such gp120-induced lysosomal exocytosis from Schwann cells induces rises in cytosolic calcium and ROS in DRG neurons. Our results suggest that gp120-induced lysosomal exocytotic release of mediators including ATP from Schwann cells through P2X4 and VNUT signaling could be a possible mechanism for HIV-associated neuropathy. The mechanism identified here may have broader impact, because many viruses and bacteria enter host cells via endocytosis (Cossart and Helenius, 2014) and some viruses have been shown to release virions via lysosomal or autophagic exocytosis (Münz, 2017). It has been shown that virus-infected cells could release ATP that activates purinergic receptors on adjacent cells (Swartz et al., 2014; Manzoor et al., 2016) and affect viral infections including HIV, hepatitis virus, influenza, and dengue viruses (Taylor and Han, 2010; Corrêa et al., 2016; Leyva-Grado et al., 2017). Similar to viruses themselves, viral proteins such as HIV-1 Tat protein has been shown to enter cells via endocytosis (Fields et al., 2015) and promote lysosome exocytosis (Fan and He, 2016). However, the potential role of virus or viral proteins in regulating purinergic receptors on lysosomes has not yet been studied. Thus, our findings provide a novel mechanism whereby viral proteins could affect lysosome purinergic receptors once entering the cell, and such a mechanism could occur in many other infectious diseases.

## Data Availability

The raw data supporting the conclusions of this manuscript will be made available by the authors, without undue reservation, to any qualified researcher.

## Author Contributions

GD, JG, and XC designed the study. GD, NM, and ZA acquired the data. GD analyzed the data and drafted the manuscript. XC contributed to interpretation of the data, revising the work of intellectual content, and final approval of the version to be published. All authors approved the final version and agreed to be accountable for all aspects of the work in ensuring that questions related to the accuracy or integrity of any part of the work were appropriately investigated and resolved.

## Conflict of Interest Statement

The authors declare that the research was conducted in the absence of any commercial or financial relationships that could be construed as a potential conflict of interest.

## References

[B1] Andrews N. W. (2000). Regulated secretion of conventional lysosomes. *Trends Cell Biol.* 10 316–321. 10.1016/s0962-8924(00)01794-3 10884683

[B2] Apostolski S. McAlarney T. Hays A. P. Latov N. (1994). Complement dependent cytotoxicity of sensory ganglion neurons mediated by the gp120 glycoprotein of HIV-1. *Immunol. Invest.* 23 47–52. 10.3109/08820139409063432 8144198

[B3] Apostolski S. McAlarney T. Quattrini A. Levison S. W. Rosoklija G. Lugaressi A. (1993). The gp120 glycoprotein of human immunodeficiency virus type 1 binds to sensory ganglion neurons. *Ann. Neurol.* 34 855–863. 10.1002/ana.410340616 8250536

[B4] Ase A. R. Honson N. S. Zaghdane H. Pfeifer T. A. Seguela P. (2015). Identification and characterization of a selective allosteric antagonist of human P2X4 receptor channels. *Mol. Pharmacol.* 87 606–616. 10.1124/mol.114.096222 25597706

[B5] Aziz-Donnelly A. Harrison T. B. (2017). Update of HIV-associated sensory neuropathies. *Curr. Treat. Options Neurol.* 19:36. 10.1007/s11940-017-0472-3 28861848

[B6] Bacallao K. Monje P. V. (2015). Requirement of cAMP signaling for schwann cell differentiation restricts the onset of myelination. *PLoS One* 10:e0116948. 10.1371/journal.pone.0116948 25705874 PMC4338006

[B7] Bachis A. Aden S. A. Nosheny R. L. Andrews P. M. Mocchetti I. (2006). Axonal transport of human immunodeficiency virus type 1 envelope protein glycoprotein 120 is found in association with neuronal apoptosis. *J. Neurosci.* 26 6771–6780. 10.1523/jneurosci.1054-06.2006 16793884 PMC6673819

[B8] Bachis A. Mocchetti I. (2004). The chemokine receptor CXCR4 and not the N-methyl-D-aspartate receptor mediates gp120 neurotoxicity in cerebellar granule cells. *J. Neurosci. Res.* 75 75–82. 10.1002/jnr.10826 14689450

[B9] Beckel J. M. Gómez N. M. Lu W. Campagno K. E. Nabet B. Albalawi F. (2018). Stimulation of TLR3 triggers release of lysosomal ATP in astrocytes and epithelial cells that requires TRPML1 channels. *Sci. Rep.* 8:5726. 10.1038/s41598-018-23877-3 29636491 PMC5893592

[B10] Beirowski B. Babetto E. Golden J. P. Chen Y. Jr. Yang K. Gross R. W. (2014). Metabolic regulator LKB1 is crucial for Schwann cell–mediated axon maintenance. *Nat. Neurosci.* 17 1351–1361. 10.1038/nn.3809 25195104 PMC4494117

[B11] Berth S. Caicedo H. H. Sarma T. Morfini G. Brady S. T. (2015). Internalization and axonal transport of the HIV glycoprotein gp120. *ASN Neuro* 7:1759091414568186. 10.1177/1759091414568186 25636314 PMC4720180

[B12] Berth S. H. Mesnard-Hoaglin N. Wang B. Kim H. Song Y. Sapar M. (2016). HIV glycoprotein Gp120 impairs fast axonal transport by activating Tak1 signaling pathways. *ASN Neuro* 8:1759091416679073. 27872270 10.1177/1759091416679073 PMC5119683

[B13] Bezzi P. Domercq M. Brambilla L. Galli R. Schols D. De Clercq E. (2001). CXCR4-activated astrocyte glutamate release via TNFalpha: amplification by microglia triggers neurotoxicity. *Nat. Neurosci.* 4 702–710. 10.1038/89490 11426226

[B14] Bilgrami M. O’Keefe P. (2014). Neurologic diseases in HIV-infected patients. *Handb. Clin. Neurol.* 121 1321–1344. 10.1016/B978-0-7020-4088-7.00090-0 24365422

[B15] Brosius Lutz A. Chung W. S. Sloan S. A. Carson G. A. Zhou L. Lovelett E. (2017). Schwann cells use TAM receptor-mediated phagocytosis in addition to autophagy to clear myelin in a mouse model of nerve injury. *Proc. Natl. Acad. Sci. U.S.A.* 114 E8072–E8080. 10.1073/pnas.1710566114 28874532 PMC5617301

[B16] Burdo T. H. Miller A. D. (2014). Animal models of HIV peripheral neuropathy. *Future Virol.* 9 465–474. 10.2217/fvl.14.28 25214880 PMC4157679

[B17] Cao Q. Zhao K. Zhong X. Z. Zou Y. Yu H. Huang P. (2014). SLC17A9 functions as a lysosomal ATP transporter and regulates cell viability. *J. Biol. Chem.* 289 23189–23199. 10.1074/jbc.M114.567107 24962569 PMC4132816

[B18] Cao Q. Zhong X. Z. Zou Y. Murrell-Lagnado R. Zhu M. X. Dong X. P. (2015). Calcium release through P2X4 activates calmodulin to promote endolysosomal membrane fusion. *J. Cell Biol.* 209 879–894. 10.1083/jcb.201409071 26101220 PMC4477861

[B19] Centner C. M. Bateman K. J. Heckmann J. M. (2013). Manifestations of HIV infection in the peripheral nervous system. *Lancet Neurol.* 12 295–309. 10.1016/S1474-4422(13)70002-4 23415569

[B20] Chen G. Zhang Z. Wei Z. Cheng Q. Li X. Li W. (2012). Lysosomal exocytosis in schwann cells contributes to axon remyelination. *Glia* 60 295–305. 10.1002/glia.21263 22042600

[B21] Cheng X. T. Xie Y. X. Zhou B. Huang N. Farfel-Becker T. Sheng Z. H. (2018). Characterization of LAMP1-labeled nondegradative lysosomal and endocytic compartments in neurons. *J. Cell Biol.* 217 3127–3139. 10.1083/jcb.201711083 29695488 PMC6123004

[B22] Chung J. M. Chung K. (2002). Importance of hyperexcitability of DRG neurons in neuropathic pain. *Pain Pract.* 2 87–97. 10.1046/j.1533-2500.2002.02011.x 17147683

[B23] Corrêa G. de Lindenberg C. A. Fernandes-Santos C. Gandini M. Petitinga Paiva F. Coutinho-Silva R. (2016). The purinergic receptor P2X7 role in control of Dengue virus-2 infection and cytokine/chemokine production in infected human monocytes. *Immunobiology* 221 794–802. 10.1016/j.imbio.2016.02.003 26969484

[B24] Cossart P. Helenius A. (2014). Endocytosis of viruses and bacteria. *Cold Spring Harb. Perspect. Biol.* 6:a016972. 10.1101/cshperspect.a016972 25085912 PMC4107984

[B25] Costantini L. M. Irvin S. C. Kennedy S. C. Guo F. Goldstein H. Herold B. C. (2015). Engineering and exploitation of a fluorescent HIV-1 gp120 for live cell CD4 binding assays. *Virology* 476 240–248. 10.1016/j.virol.2014.12.019 25555152 PMC4323844

[B26] Deng H. Liu R. Ellmeier W. Choe S. Unutmaz D. Burkhart M. (1996). Identification of a major co-receptor for primary isolates of HIV-1. *Nature* 381 661–666. 10.1038/381661a0 8649511

[B27] Dong X. (2015). P2X4 forms ATP-activated channels on lysosomal membranes regulated by luminal pH and SLC17A9 proteins. *Biophys. J.* 108:419a 10.1016/j.bpj.2014.11.2293 PMC4067200 24817123

[B28] Eitan E. Suire C. Zhang S. Mattson M. P. (2016). Impact of lysosome status on extracellular vesicle content and release. *Ageing Res. Rev.* 32 65–74. 10.1016/j.arr.2016.05.001 27238186 PMC5154730

[B29] Ellis R. J. Marquie-Beck J. Delaney P. Alexander T. Clifford D. B. McArthur J. C. (2008). Human immunodeficiency virus protease inhibitors and risk for peripheral neuropathy. *Ann. Neurol.* 64 566–572. 10.1002/ana.21484 19067367 PMC2605176

[B30] Emmett D. S. Feranchak A. Kilic G. Puljak L. Miller B. Dolovcak S. (2008). Characterization of ionotrophic purinergic receptors in hepatocytes. *Hepatology* 47 698–705. 10.1002/hep.22035 18027885

[B31] Fan Y. He J. J. (2016). HIV-1 tat promotes lysosomal exocytosis in astrocytes and contributes to astrocyte-mediated tat neurotoxicity. *J. Biol. Chem.* 291 22830–22840. 10.1074/jbc.m116.731836 27609518 PMC5077215

[B32] Feldman E. L. Nave K. A. Jensen T. S. Bennett D. L. H. (2017). New horizons in diabetic neuropathy: mechanisms, bioenergetics, and pain. *Neuron* 93 1296–1313. 10.1016/j.neuron.2017.02.005 28334605 PMC5400015

[B33] Feng Y. Press B. Wandinger-Ness A. (1995). Rab 7: an important regulator of late endocytic membrane traffic. *J. Cell Biol.* 131 1435–1452. 10.1083/jcb.131.6.1435 8522602 PMC2120682

[B34] Fields J. Dumaop W. Elueteri S. Campos S. Serger E. Trejo M. (2015). HIV-1 tat alters neuronal autophagy by modulating autophagosome fusion to the lysosome: implications for HIV-associated neurocognitive disorders. *J. Neurosci.* 35 1921–1938. 10.1523/JNEUROSCI.3207-14.2015 25653352 PMC4315828

[B35] Fuller G. N. Jacobs J. M. Guiloff R. J. (1993). Nature and incidence of peripheral nerve syndromes in HIV infection. *J. Neurol. Neurosurg. Psychiatry* 56 372–381. 10.1136/jnnp.56.4.372 8387098 PMC1014954

[B36] Gelderblom H. R. Hausmann E. H. Ozel M. Pauli G. Koch M. A. (1987). Fine structure of human immunodeficiency virus (HIV) and immunolocalization of structural proteins. *Virology* 156 171–176. 10.1016/0042-6822(87)90449-1 3643678

[B37] Gregg E. W. Sorlie P. Paulose-Ram R. Gu Q. Eberhardt M. S. Wolz M. (2004). Prevalence of lower-extremity disease in the US adult population >=40 years of age with and without diabetes: 1999-2000 national health and nutrition examination survey. *Diabetes Care* 27 1591–1597. 10.2337/diacare.27.7.1591 15220233

[B38] Guha S. Baltazar G. C. Coffey E. E. Tu L. A. Lim J. C. Beckel J. M. (2013). Lysosomal alkalinization, lipid oxidation, and reduced phagosome clearance triggered by activation of the P2X7 receptor. *FASEB J.* 27 4500–4509. 10.1096/fj.13-236166 23964074 PMC3804754

[B39] Hall C. D. Snyder C. R. Messenheimer J. A. Wilkins J. W. Robertson W. T. Whaley R. A. (1991). Peripheral neuropathy in a cohort of human immunodeficiency virus-infected patients. Incidence and relationship to other nervous system dysfunction. *Arch. Neurol.* 48 1273–1274. 1668978 10.1001/archneur.1991.00530240077026

[B40] Hao S. (2013). The molecular and pharmacological mechanisms of HIV-related neuropathic pain. *Curr. Neuropharmacol.* 11 499–512. 10.2174/1570159X11311050005 24403874 PMC3763758

[B41] Harada A. Takei Y. Kanai Y. Tanaka Y. Nonaka S. Hirokawa N. (1998). Golgi vesiculation and lysosome dispersion in cells lacking cytoplasmic dynein. *J. Cell Biol.* 141 51–59. 10.1083/jcb.141.1.51 9531547 PMC2132725

[B42] Hattori M. Gouaux E. (2012). Molecular mechanism of ATP binding and ion channel activation in P2X receptors. *Nature* 485 207–212. 10.1038/nature11010 22535247 PMC3391165

[B43] Herzberg U. Sagen J. (2001). Peripheral nerve exposure to HIV viral envelope protein gp120 induces neuropathic pain and spinal gliosis. *J. Neuroimmunol.* 116 29–39. 10.1016/s0165-5728(01)00288-0 11311327

[B44] Hoke A. Morris M. Haughey N. J. (2009). GPI-1046 protects dorsal root ganglia from gp120-induced axonal injury by modulating store-operated calcium entry. *J. Peripher. Nerv. Syst.* 14 27–35. 10.1111/j.1529-8027.2009.00203.x 19335537 PMC2728770

[B45] Hollenbeck P. J. Swanson J. A. (1990). Radial extension of macrophage tubular lysosomes supported by kinesin. *Nature* 346 864–866. 10.1038/346864a0 1697403

[B46] Huang P. Zou Y. Zhong X. Z. Cao Q. Zhao K. Zhu M. X. (2014). P2X4 forms functional ATP-activated cation channels on lysosomal membranes regulated by luminal pH. *J. Biol. Chem.* 289 17658–17667. 10.1074/jbc.M114.552158 24817123 PMC4067200

[B47] Iida T. Yi H. Liu S. Huang W. Kanda H. Lubarsky D. A. (2016). Spinal CPEB-mtROS-CBP signaling pathway contributes to perineural HIV gp120 with ddC-related neuropathic pain in rats. *Exp. Neurol.* 281 17–27. 10.1016/j.expneurol.2016.04.012 27090160

[B48] Ino D. Sagara H. Suzuki J. Kanemaru K. Okubo Y. Iino M. (2015). Neuronal regulation of schwann cell mitochondrial Ca(2+) signaling during myelination. *Cell Rep.* 12 1951–1959. 10.1016/j.celrep.2015.08.039 26365190

[B49] Jaiswal J. K. Andrews N. W. Simon S. M. (2002). Membrane proximal lysosomes are the major vesicles responsible for calcium-dependent exocytosis in nonsecretory cells. *J. Cell Biol.* 159 625–635. 10.1083/jcb.200208154 12438417 PMC2173094

[B50] Jang S. Y. Yoon B. A. Shin Y. K. Yun S. H. Jo Y. R. Choi Y. Y. (2017). Schwann cell dedifferentiation-associated demyelination leads to exocytotic myelin clearance in inflammatory segmental demyelination. *Glia* 65 1848–1862. 10.1002/glia.23200 28795433

[B51] Jessen K. R. Mirsky R. (2016). The repair schwann cell and its function in regenerating nerves. *J. Physiol.* 594 3521–3531. 10.1113/JP270874 26864683 PMC4929314

[B52] Jia L. Chopp M. Wang L. Lu X. Szalad A. Zhang Z. G. (2018). Exosomes derived from high-glucose–stimulated Schwann cells promote development of diabetic peripheral neuropathy. *FASEB J.* 32 6911–6922. 10.1096/fj.201800597R 29932869 PMC6219828

[B53] Jia R. Guardia C. M. Pu J. Chen Y. Bonifacino J. S. (2017). BORC coordinates encounter and fusion of lysosomes with autophagosomes. *Autophagy* 13 1648–1663. 10.1080/15548627.2017.1343768 28825857 PMC5640200

[B54] Johansson M. Rocha N. Zwart W. Jordens I. Janssen L. Kuijl C. (2007). Activation of endosomal dynein motors by stepwise assembly of Rab7–RILP–p150Glued, ORP1L, and the receptor βlll spectrin. *J. Cell Biol.* 176 459–471. 10.1083/jcb.200606077 17283181 PMC2063981

[B55] Johnson D. E. Ostrowski P. Jaumouillé V. Grinstein S. (2016). The position of lysosomes within the cell determines their luminal pH. *J. Cell Biol.* 212 677–692. 10.1083/jcb.201507112 26975849 PMC4792074

[B56] Jordens I. Fernandez-Borja M. Marsman M. Dusseljee S. Janssen L. Calafat J. (2001). The Rab7 effector protein RILP controls lysosomal transport by inducing the recruitment of dynein-dynactin motors. *Curr. Biol.* 11 1680–1685. 10.1016/s0960-9822(01)00531-0 11696325

[B57] Jung J. Jo H. W. Kwon H. Jeong N. Y. (2014). ATP release through lysosomal exocytosis from peripheral nerves: the effect of lysosomal exocytosis on peripheral nerve degeneration and regeneration after nerve injury. *Biomed. Res. Int.* 2014:936891. 10.1155/2014/936891 25101301 PMC4101216

[B58] Jurga A. M. Piotrowska A. Makuch W. Przewlocka B. Mika J. (2017). Blockade of P2X4 receptors inhibits neuropathic pain-related behavior by preventing MMP-9 activation and, consequently, pronociceptive interleukin release in a rat model. *Front. Pharmacol.* 8:48. 10.3389/fphar.2017.00048 28275350 PMC5321202

[B59] Kaku M. Simpson D. M. (2014). HIV neuropathy. *Curr. Opin. HIV AIDS* 9 521–526. 10.1097/COH.0000000000000103 25275705

[B60] Kato Y. Hiasa M. Ichikawa R. Hasuzawa N. Kadowaki A. Iwatsuki K. (2017). Identification of a vesicular ATP release inhibitor for the treatment of neuropathic and inflammatory pain. *Proc. Natl. Acad. Sci. U.S.A.* [Epub ahead of print]. 28720702 10.1073/pnas.1704847114 PMC5547629

[B61] Kaul M. Lipton S. A. (1999). Chemokines and activated macrophages in HIV gp120-induced neuronal apoptosis. *Proc. Natl. Acad. Sci. U.S.A.* 96 8212–8216. 10.1073/pnas.96.14.8212 10393974 PMC22214

[B62] Keswani S. C. Polley M. Pardo C. A. Griffin J. W. McArthur J. C. Hoke A. (2003). Schwann cell chemokine receptors mediate HIV-1 gp120 toxicity to sensory neurons. *Ann. Neurol.* 54 287–296. 10.1002/ana.10645 12953261

[B63] Kim S. Maynard J. C. Strickland A. Burlingame A. L. Milbrandt J. (2018). Schwann cell O-GlcNAcylation promotes peripheral nerve remyelination via attenuation of the AP-1 transcription factor JUN. *Proc. Natl. Acad. Sci. U.S.A.* 115 8019–8024. 10.1073/pnas.1805538115 30012597 PMC6077742

[B64] Korolchuk V. I. Saiki S. Lichtenberg M. Siddiqi F. H. Roberts E. A. Imarisio S. (2011). Lysosomal positioning coordinates cellular nutrient responses. *Nat. Cell Biol.* 13 453–460. 10.1038/ncb2204 21394080 PMC3071334

[B65] Kostyuk E. Voitenko N. Kruglikov I. Shmigol A. Shishkin V. Efimov A. (2001). Diabetes-induced changes in calcium homeostasis and the effects of calcium channel blockers in rat and mice nociceptive neurons. *Diabetologia* 44 1302–1309. 10.1007/s001250100642 11692179

[B66] Langemeyer L. Fröhlich F. Ungermann C. (2018). Rab GTPase function in endosome and lysosome biogenesis. *Trends Cell Biol.* 28 957–970. 10.1016/j.tcb.2018.06.007 30025982

[B67] Layne S. P. Merges M. J. Dembo M. Spouge J. L. Conley S. R. Moore J. P. (1992). Factors underlying spontaneous inactivation and susceptibility to neutralization of human immunodeficiency virus. *Virology* 189 695–714. 10.1016/0042-6822(92)90593-e 1386485

[B68] Lehmann H. C. Chen W. Borzan J. Mankowski J. L. Hoke A. (2011). Mitochondrial dysfunction in distal axons contributes to human immunodeficiency virus sensory neuropathy. *Ann. Neurol.* 69 100–110. 10.1002/ana.22150 21280080 PMC3051401

[B69] Letendre S. L. Ellis R. J. Everall I. Ances B. Bharti A. McCutchan J. A. (2009). Neurologic complications of HIV disease and their treatment. *Top. HIV Med.* 17 46–56. 19401607 PMC3065886

[B70] Leyva-Grado V. H. Ermler M. E. Schotsaert M. Gonzalez M. G. Lim J. K. García-Sastre A. (2017). Contribution of the purinergic receptor P2X7 to development of lung immunopathology during influenza virus infection. *mBio* 8:e229-17. 10.1128/mBio.00229-17 28351919 PMC5371412

[B71] Li D. Ropert N. Koulakoff A. Giaume C. Oheim M. (2008). Lysosomes are the major vesicular compartment undergoing Ca2+-regulated exocytosis from cortical astrocytes. *J. Neurosci.* 28 7648–7658. 10.1523/JNEUROSCI.0744-08.2008 18650341 PMC6670856

[B72] Linke M. Herzog V. Brix K. (2002). Trafficking of lysosomal cathepsin B—green fluorescent protein to the surface of thyroid epithelial cells involves the endosomal/lysosomal compartment. *J. Cell Sci.* 115 4877–4889. 10.1242/jcs.00184 12432075

[B73] Liu T. Sun L. Xiong Y. Shang S. Guo N. Teng S. (2011). Calcium triggers exocytosis from two types of organelles in a single astrocyte. *J. Neurosci.* 31 10593–10601. 10.1523/JNEUROSCI.6401-10.2011 21775603 PMC6622647

[B74] Lopez-Leal R. Court F. A. (2016). Schwann cell exosomes mediate neuron-glia communication and enhance axonal regeneration. *Cell Mol. Neurobiol.* 36 429–436. 10.1007/s10571-015-0314-3 26993502 PMC11482438

[B75] Lopez-Verrilli M. A. Court F. A. (2012). Transfer of vesicles from schwann cells to axons: a novel mechanism of communication in the peripheral nervous system. *Front. Physiol.* 3:205. 10.3389/fphys.2012.00205 22707941 PMC3374349

[B76] Manzoor S. Akhtar U. Naseem S. Khalid M. Mazhar M. Parvaiz F. (2016). Ionotropic purinergic receptors P2X4 and P2X7: proviral or antiviral? An insight into P2X receptor signaling and hepatitis C virus infection. *Viral Immunol.* 29 401–408. 10.1089/vim.2016.0008 27526181

[B77] Martinez I. Chakrabarti S. Hellevik T. Morehead J. Fowler K. Andrews N. W. (2000). Synaptotagmin VII regulates Ca(2+) dependent exocytosis of lysosomes in fibroblasts. *J. Cell Biol.* 148 1141–1150. 10.1083/jcb.148.6.1141 10725327 PMC2174306

[B78] Martyn C. N. Hughes R. A. (1997). Epidemiology of peripheral neuropathy. *J. Neurol. Neurosurg. Psychiatry* 62 310–318. 9120441 10.1136/jnnp.62.4.310 PMC1074084

[B79] Masuda T. Ozono Y. Mikuriya S. Kohro Y. Tozaki-Saitoh H. Iwatsuki K. (2016). Dorsal horn neurons release extracellular ATP in a VNUT-dependent manner that underlies neuropathic pain. *Nat. Commun.* 7:12529. 10.1038/ncomms12529 27515581 PMC4990655

[B80] Materazzi S. Fusi C. Benemei S. Pedretti P. Patacchini R. Nilius B. (2012). TRPA1 and TRPV4 mediate paclitaxel-induced peripheral neuropathy in mice via a glutathione-sensitive mechanism. *Pflügers Arch.* 463 561–569. 10.1007/s00424-011-1071-x 22258694

[B81] Medina D. L. Fraldi A. Bouche V. Annunziata F. Mansueto G. Spampanato C. (2011). Transcriptional activation of lysosomal exocytosis promotes cellular clearance. *Dev. Cell* 21 421–430. 10.1016/j.devcel.2011.07.016 21889421 PMC3173716

[B82] Melli G. Keswani S. C. Fischer A. Chen W. Hoke A. (2006). Spatially distinct and functionally independent mechanisms of axonal degeneration in a model of HIV-associated sensory neuropathy. *Brain* 129(Pt 5), 1330–1338. 10.1093/brain/awl058 16537566

[B83] Meresse S. Gorvel J. P. Chavrier P. (1995). The rab7 GTPase resides on a vesicular compartment connected to lysosomes. *J. Cell Sci.* 108 3349–3358. 8586647 10.1242/jcs.108.11.3349

[B84] Monk K. R. Feltri M. L. Taveggia C. (2015). New insights on schwann cell development. *Glia* 63 1376–1393. 10.1002/glia.22852 25921593 PMC4470834

[B85] Moriyama Y. Hiasa M. Sakamoto S. Omote H. Nomura M. (2017). Vesicular nucleotide transporter (VNUT): appearance of an actress on the stage of purinergic signaling. *Purinergic Signal* 13 387–404. 10.1007/s11302-017-9568-1 28616712 PMC5563297

[B86] Moriyama Y. Nomura M. (2017). Clodronate: a vesicular ATP release blocker. *Trends Pharmacol. Sci.* 39 13–23. 10.1016/j.tips.2017.10.007 29146440

[B87] Münz C. (2017). The autophagic machinery in viral exocytosis. *Front. Microbiol.* 8:269. 10.3389/fmicb.2017.00269 28270807 PMC5318907

[B88] Murrell-Lagnado R. Robinson L. (2013). The trafficking and targeting of P2X receptors. *Front. Cell. Neurosci.* 7:233. 10.3389/fncel.2013.00233 24319412 PMC3837535

[B89] Nagano I. Shapshak P. Yoshioka M. Xin K. Nakamura S. Bradley W. G. (1996). Increased NADPH-diaphorase reactivity and cytokine expression in dorsal root ganglia in acquired immunodeficiency syndrome. *J. Neurol. Sci.* 136 117–128. 10.1016/0022-510x(95)00317-u 8815158

[B90] Nobbio L. Sturla L. Fiorese F. Usai C. Basile G. Moreschi I. (2009). P2X7-mediated increased intracellular calcium causes functional derangement in schwann cells from rats with CMT1A neuropathy. *J. Biol. Chem.* 284 23146–23158. 10.1074/jbc.M109.027128 19546221 PMC2755720

[B91] Norrmén C. Figlia G. Pfistner P. Pereira J. A. Bachofner S. Suter U. (2018). mTORC1 Is transiently reactivated in injured nerves to Promote c-Jun elevation and schwann cell dedifferentiation. *J. Neurosci.* 38 4811–4828. 10.1523/JNEUROSCI.3619-17.2018 29695414 PMC5956991

[B92] Oh S. B. Tran P. B. Gillard S. E. Hurley R. W. Hammond D. L. Miller R. J. (2001). Chemokines and glycoprotein120 produce pain hypersensitivity by directly exciting primary nociceptive neurons. *J. Neurosci.* 21 5027–5035. 10.1523/jneurosci.21-14-05027.2001 11438578 PMC6762869

[B93] Oh S. K. Cruikshank W. W. Raina J. Blanchard G. C. Adler W. H. Walker J. (1992). Identification of HIV-1 envelope glycoprotein in the serum of AIDS and ARC patients. *J. Acquir. Immune Defic. Syndr.* 5 251–256. 1740750

[B94] Orita S. Henry K. Mantuano E. Yamauchi K. De Corato A. Ishikawa T. (2013). Schwann Cell LRP1 regulates remak bundle ultrastructure and axonal interactions to prevent neuropathic pain. *J. Neurosci.* 33 5590–5602. 10.1523/JNEUROSCI.3342-12.2013 23536074 PMC3837698

[B95] Popovic M. Tenner-Racz K. Pelser C. Stellbrink H. J. van Lunzen J. Lewis G. (2005). Persistence of HIV-1 structural proteins and glycoproteins in lymph nodes of patients under highly active antiretroviral therapy. *Proc. Natl. Acad. Sci. U.S.A.* 102 14807–14812. 10.1073/pnas.0506857102 16199516 PMC1253583

[B96] Prior D. E. Song N. Cohen J. A. (2018). Neuromuscular diseases associated with human immunodeficiency virus infection. *J. Neurol. Sci.* 387 27–36. 10.1016/j.jns.2018.01.016 29571868

[B97] Progida C. Bakke O. (2016). Bidirectional traffic between the golgi and the endosomes – machineries and regulation. *J. Cell Sci.* 129 3971–3982. 27802132 10.1242/jcs.185702

[B98] Qi J. Buzas K. Fan H. Cohen J. I. Wang K. Mont E. (2011). Painful pathways induced by TLR stimulation of dorsal root ganglion neurons. *J. Immunol.* 186 6417–6426. 10.4049/jimmunol.1001241 21515789 PMC3098909

[B99] Rodella U. Negro S. Scorzeto M. Bergamin E. Jalink K. Montecucco C. (2017). Schwann cells are activated by ATP released from neurons in an in vitro cellular model of miller fisher syndrome. *Dis. Model. Mech.* 10 597–603. 10.1242/dmm.027870 28067631 PMC5451166

[B100] Rodríguez A. Webster P. Ortego J. Andrews N. W. (1997). Lysosomes behave as Ca2+ regulated exocytic vesicles in fibroblasts and epithelial cells. *J. Cell Biol.* 137 93–104. 10.1083/jcb.137.1.93 9105039 PMC2139854

[B101] Rychert J. Strick D. Bazner S. Robinson J. Rosenberg E. (2010). Detection of HIV gp120 in plasma during early HIV infection is associated with increased proinflammatory and immunoregulatory cytokines. *AIDS Res. Hum. Retroviruses* 26 1139–1145. 10.1089/aid.2009.0290 20722464 PMC2982714

[B102] Salvemini D. Little J. W. Doyle T. Neumann W. L. (2011). Roles of reactive oxygen and nitrogen species in pain. *Free Radic. Biol. Med.* 51 951–966. 10.1016/j.freeradbiomed.2011.01.026 21277369 PMC3134634

[B103] Santosuosso M. Righi E. Lindstrom V. Leblanc P. R. Poznansky M. C. (2009). HIV-1 envelope protein gp120 is present at high concentrations in secondary lymphoid organs of individuals with chronic HIV-1 infection. *J. Infect. Dis.* 200 1050–1053. 10.1086/605695 19698075

[B104] Sasaki Y. Hackett A. R. Kim S. Strickland A. Milbrandt J. (2018). Dysregulation of NAD(+) metabolism induces a schwann cell dedifferentiation program. *J. Neurosci.* 38 6546–6562. 10.1523/JNEUROSCI.3304-17.2018 29921717 PMC6052240

[B105] Schütz S. G. Robinson-Papp J. (2013). HIV-related neuropathy: current perspectives. *HIVAIDS* 5 243–251. 10.2147/HIV.S36674 24049460 PMC3775622

[B106] Shin Y. H. Chung H.-J. Park C. Jung J. Jeong N. Y. Neurobiology M. (2014). Adenosine 5’-Triphosphate (ATP) inhibits schwann cell demyelination during wallerian degeneration. *Cell Mol. Neurobiol.* 34 361–368. 10.1007/s10571-013-0020-y 24363123 PMC11488926

[B107] Shin Y. H. Lee S. J. Jung J. (2012). Secretion of ATP from schwann cells through lysosomal exocytosis during wallerian degeneration. *Biochem. Biophys. Res. Commun.* 429 163–167. 10.1016/j.bbrc.2012.10.121 23142593

[B108] Sivaramakrishnan V. Bidula S. Campwala H. Katikaneni D. Fountain S. J. (2012). Constitutive lysosome exocytosis releases ATP and engages P2Y receptors in human monocytes. *J. Cell Sci.* 125 4567–4575. 10.1242/jcs.107318 22767503

[B109] Smart M. L. Gu B. Panchal R. G. Wiley J. Cromer B. Williams D. A. (2003). P2X7 receptor cell surface expression and cytolytic pore formation are regulated by a distal C-terminal region. *J. Biol. Chem.* 278 8853–8860. 10.1074/jbc.m211094200 12496266

[B110] Soto J. Monje P. V. (2017). Axon contact-driven Schwann cell dedifferentiation. *Glia* 65 864–882. 10.1002/glia.23131 28233923 PMC5395415

[B111] Stavros K. Simpson D. M. (2014). Understanding the etiology and management of HIV-associated peripheral neuropathy. *Curr. HIVAIDS Rep.* 11 195–201. 10.1007/s11904-014-0211-2 24969360

[B112] Stevens B. Fields R. D. (2000). Response of schwann cells to action potentials in development. *Science* 287 2267–2271. 10.1126/science.287.5461.2267 10731149

[B113] Stokes L. Scurrah K. Ellis J. A. Cromer B. A. Skarratt K. K. Gu B. J. (2011). A loss-of-function polymorphism in the human P2X4 receptor is associated with increased pulse pressure. *Hypertension* 58 1086–1092. 10.1161/HYPERTENSIONAHA.111.176180 22068874

[B114] Su W. F. Wu F. Jin Z. H. Gu Y. Chen Y. T. Fei Y. (2019). Overexpression of P2X4 receptor in Schwann cells promotes motor and sensory functional recovery and remyelination via BDNF secretion after nerve injury. *Glia* 67 78–90. 10.1002/glia.23527 30306657

[B115] Surprenant A. Rassendren F. Kawashima E. North R. A. Buell G. (1996). The cytolytic P2Z receptor for extracellular ATP identified as a P2X receptor (P2X7). *Science* 272 735–738. 10.1126/science.272.5262.735 8614837

[B116] Swartz T. H. Esposito A. M. Durham N. D. Hartmann B. M. Chen B. K. (2014). P2X-selective purinergic antagonists are strong inhibitors of HIV-1 fusion during both cell-to-cell and cell-free infection. *J. Virol.* 88 11504–11515. 10.1128/JVI.01158-14 25031337 PMC4178786

[B117] Takenouchi T. Nakai M. Iwamaru Y. Sugama S. Tsukimoto M. Fujita M. (2009). The activation of P2X7 receptor impairs lysosomal functions and stimulates the release of autophagolysosomes in microglial cells. *J. Immunol.* 182 2051–2062. 10.4049/jimmunol.0802577 19201858

[B118] Taylor J. M. Han Z. (2010). Purinergic receptor functionality is necessary for infection of human hepatocytes by hepatitis delta virus and hepatitis B virus. *PLoS One* 5:e15784. 10.1371/journal.pone.0015784 21187936 PMC3004961

[B119] Tucker W. C. Weber T. Chapman E. R. (2004). Reconstitution of Ca2+-regulated membrane fusion by synaptotagmin and SNAREs. *Science* 304 435–438. 10.1126/science.1097196 15044754

[B120] Viader A. Sasaki Y. Kim S. Strickland A. Cayce Workman S. Yang K. (2013). Aberrant schwann cell lipid metabolism linked to mitochondrial deficits leads to axon degeneration and neuropathy. *Neuron* 77 886–898. 10.1016/j.neuron.2013.01.012 23473319 PMC3594792

[B121] Wallace V. C. Blackbeard J. Pheby T. Segerdahl A. R. Davies M. Hasnie F. (2007). Pharmacological, behavioural and mechanistic analysis of HIV-1 gp120 induced painful neuropathy. *Pain* 133 47–63. 10.1016/j.pain.2007.02.015 17433546 PMC2706950

[B122] Wenzel E. D. Bachis A. Avdoshina V. Taraballi F. Tasciotti E. Mocchetti I. (2017). Endocytic trafficking of HIV gp120 is mediated by dynamin and plays a role in gp120 neurotoxicity. *J. Neuroimmune Pharmacol.* 12 492–503. 10.1007/s11481-017-9739-4 28349243 PMC5815508

[B123] Wyatt R. Sodroski J. (1998). The HIV-1 envelope glycoproteins: fusogens, antigens, and immunogens. *Science* 280 1884–1888. 10.1126/science.280.5371.1884 9632381

[B124] Xu J. Chai H. Ehinger K. Egan T. M. Srinivasan R. Frick M. (2014). Imaging P2X4 receptor subcellular distribution, trafficking, and regulation using P2X4-pHluorin. *J. Gen. Physiol.* 144 81–104. 10.1085/jgp.201411169 24935743 PMC4076521

[B125] Yamashita T. Yamamoto S. Zhang J. Kometani M. Tomiyama D. Kohno K. (2016). Duloxetine inhibits microglial P2X4 receptor function and alleviates neuropathic pain after peripheral nerve injury. *PLoS One* 11:e0165189. 10.1371/journal.pone.0165189 27768754 PMC5074465

[B126] Yang J. Xie M. X. Hu L. Wang X. F. Mai J. Z. Li Y. Y. (2018). Upregulation of N-type calcium channels in the soma of uninjured dorsal root ganglion neurons contributes to neuropathic pain by increasing neuronal excitability following peripheral nerve injury. *Brain Behav. Immun.* 71 52–65. 10.1016/j.bbi.2018.04.016 29709527

[B127] Yeh T. M. Evans S. R. Gulick R. M. Clifford D. B. (2010). Vicriviroc and peripheral neuropathy: results from AIDS Clinical Trials Group 5211. *HIV Clin. Trials* 11 51–58. 10.1310/hct1101-51 20400411 PMC2958041

[B128] Ying M. Liu H. Zhang T. Jiang C. Gong Y. Wu B. (2017). Effect of artemisinin on neuropathic pain mediated by P2X4 receptor in dorsal root ganglia. *Neurochem. Int.* 108 27–33. 10.1016/j.neuint.2017.02.004 28192150

[B129] Yuan S. B. Shi Y. Chen J. Zhou X. Li G. Gelman B. B. (2014). Gp120 in the pathogenesis of human immunodeficiency virus–associated pain. *Ann. Neurol.* 75 837–850. 10.1002/ana.24139 24633867 PMC4077969

[B130] Zhang Z. Chen G. Zhou W. Song A. Xu T. Luo Q. (2007). Regulated ATP release from astrocytes through lysosome exocytosis. *Nat. Cell Biol.* 9 945–953. 10.1038/ncb1620 17618272

[B131] Zhong X. Z. Cao Q. Sun X. Dong X. P. (2016). Activation of lysosomal P2X4 by ATP transported into lysosomes via VNUT/SLC17A9 using V-ATPase generated voltage gradient as the driving force. *J. Physiol.* 594 4253–4266. 10.1113/JP271893 27477609 PMC4967729

[B132] Zhou M. Hu M. He S. Li B. Liu C. Min J. (2018). Effects of RSC96 schwann cell-derived exosomes on proliferation, senescence, and apoptosis of dorsal root ganglion cells *In Vitro*. *Med. Sci. Monit.* 24 7841–7849. 10.12659/MSM.909509 30387453 PMC6228118

