# Supplementary material for: HIV-1 gp120 Promotes Lysosomal Exocytosis in Human Schwann Cells
Source: Front Cell Neurosci. 2019 Jul 17;13:329. doi: 10.3389/fncel.2019.00329 (PMC6650616; doi:10.3389/fncel.2019.00329)
Supplement: Supplementary file 1 [file Data_Sheet_1.pdf]

Supplementary Figure S1

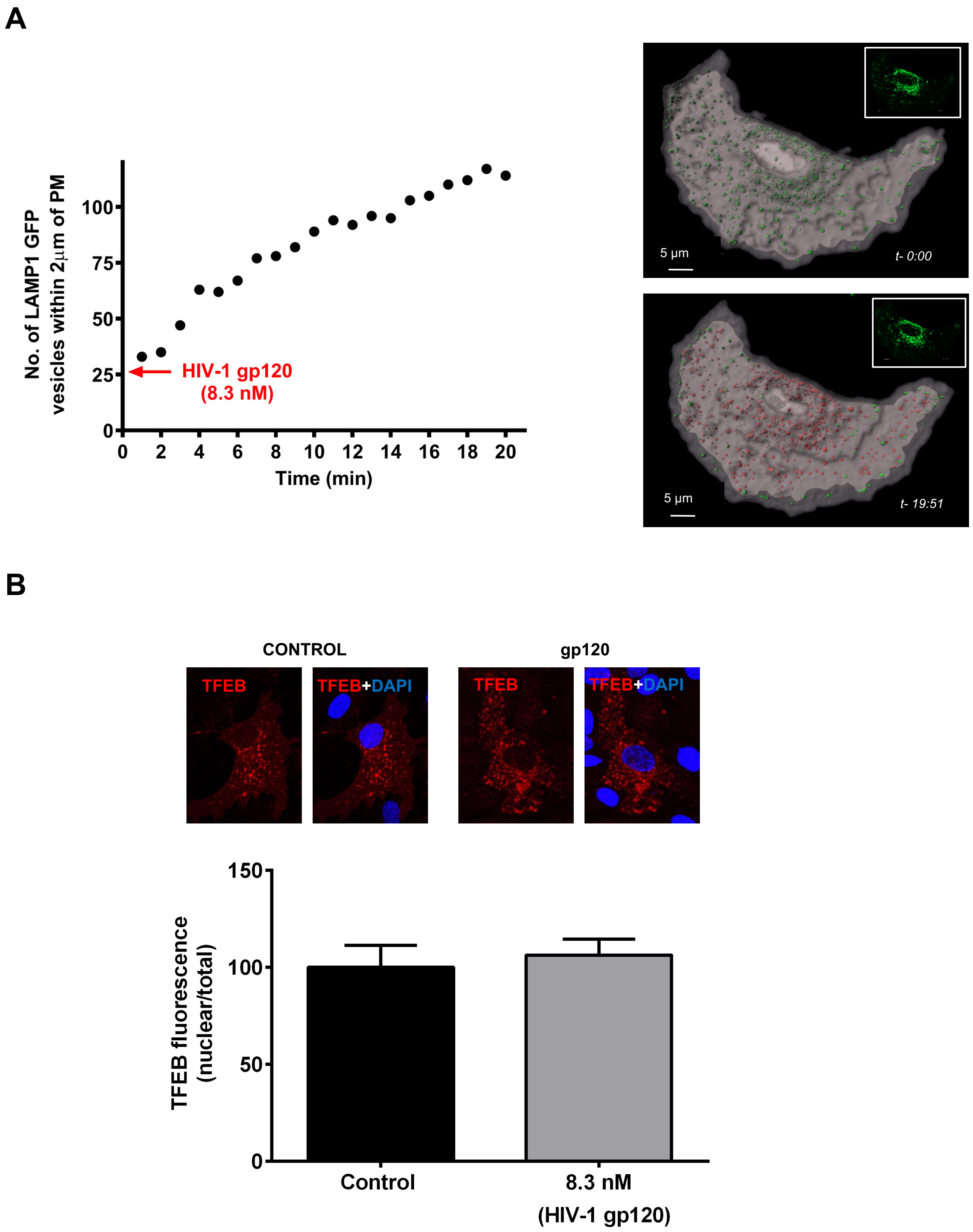

Figure S1. (A) As shown in the representative time-lapse imaging, where peripheral lysosomes are labelled green and juxtanuclear lysosomes are labelled red, gp120 treatment (8.3 nM for 40 min), in a time-dependent manner, increased the number of LAMP1-GFP vesicles within 2 μm of the plasma membrane (PM) of hSCs (bar= 5 μm). (B) As shown in representative confocal images of hScs immunostained with TFEB antibodies (red) and DAPI for nuclei (blue), gp120 treatment (8.3 nM for 40 min) did not change significantly nuclear translocation of TFEB (n=30, p=0.4803)

Supplementary Figure S2

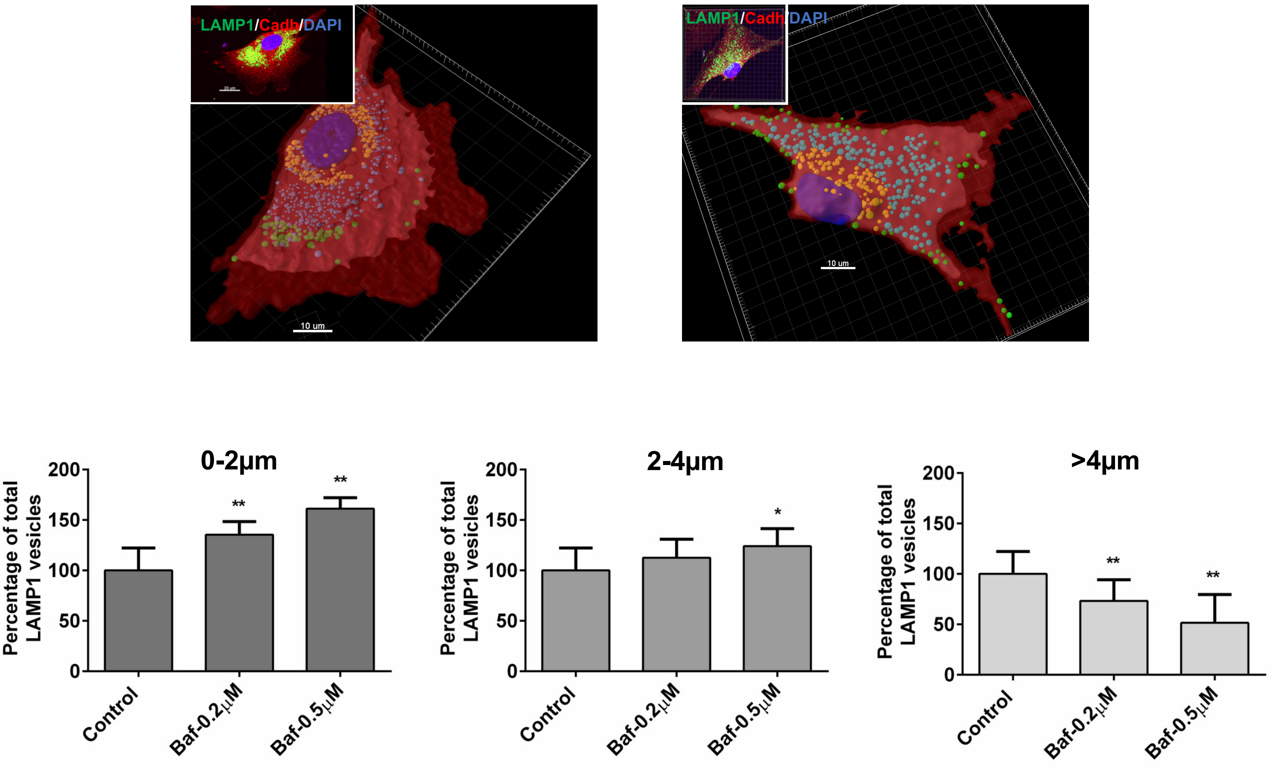

Figure S2. As shown in representative LAMP1 staining (insert) and 3D reconstructed images, bafilomycin (Baf, 0.2-0.5 μM, for 40 min) increased the percentage of peripheral (green) and juxtanuclear (magenta) lysosomes and decreased the percentage of perinuclear (yellow) lysosomes (n=3, *p<0.05). Plasma membrane were outlined with Pan-Cadherin (red) and the nucleus stained with DAPI (blue). Classification scheme of LAMP1 positive lysosomes in concentric shells labeled as peripheral (0-2 μm from PM) , juxtanuclear (2-4 μm from PM), and perinuclear (>4 μm from PM) lysosomes.

Supplementary Figure S3

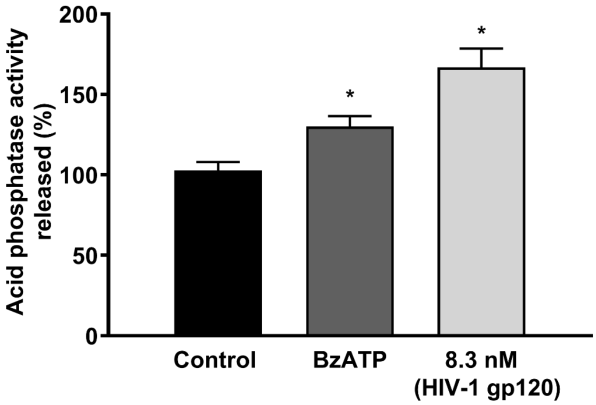

F**igure S3.** Similar to gp120 (to a lesser extent), the P2X4 agonist BzATP (5.0 µM for 40 min) increased the activity of acid phosphatase release in media (n=3, *p<0.05).

Supplementary Figure S4

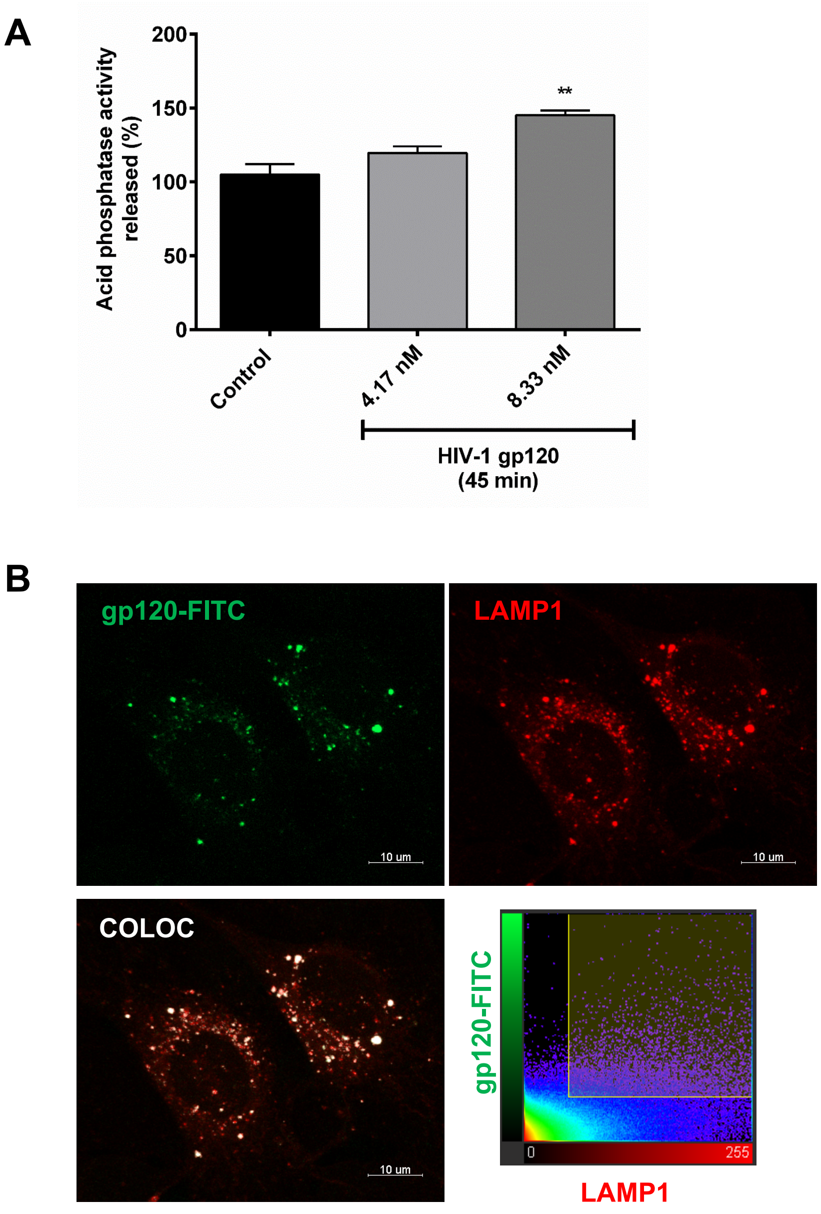

Figure S4. (A) As compared with heat-inactivated gp120 (control), gp120 treatment for 45 min significantly increased the release of lysosomal acid phosphatase in media of rat schwannoma RT4 cells (n=3, **p<0.01). (B) Representative confocal images show the presence of gp120-FITC in lysosomes (LAMP1-RFP) in hSCs following 30 min treatment of gp120 FITC. Corresponding scatterplot shows that Pearson's coefficient is 0.456.

Supplementary Figure S5

A

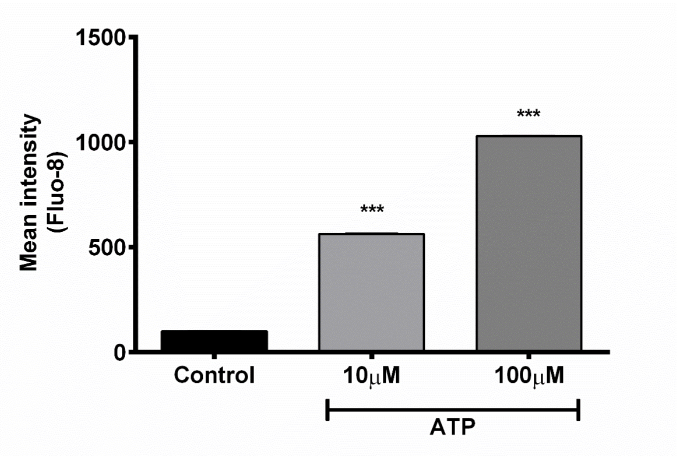

B

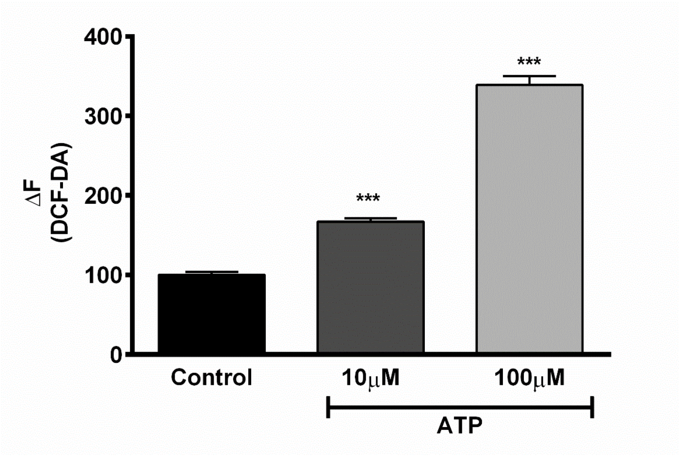

F**igure S5.** (A) ATP (10 and 100μM) added directly to DRG neurons increased intracellular Ca^{2+} as measured by Fluo-8 (n=3, ***p<0.01). (B) ATP (10 and 100μM) added directly to DRG neurons increase in cytosolic ROS as measured by DCF-DA (n=3, ***p<0.01).
